# Functional Food Potential of *Magnolia liliiflora* Leaves: Chemical Profiling of Bioactive Lignans and Their Anti-Inflammatory Effects in LPS-Activated Microglia

**DOI:** 10.3390/nu18111749

**Published:** 2026-05-29

**Authors:** Jorge-Eduardo Ponce-Zea, Yun-Hui Che, Gwan-Young Jung, Van-Hieu Mai, Minh-Thi-Tuyet Le, Jin-Pyo An, Won-Keun Oh

**Affiliations:** 1 Research Institute of Pharmaceutical Sciences, College of Pharmacy, Seoul National University, Seoul 08826, Republic of Korea; jepz210689@snu.ac.kr (J.-E.P.-Z.); woonhye419@snu.ac.kr (Y.-H.C.); rhksdud9951@snu.ac.kr (G.-Y.J.); maihieu@snu.ac.kr (V.-H.M.); lethituyetminh19289@gmail.com (M.-T.-T.L.); 2 Department of Industrial Crop Science and Technology, College of Agriculture, Life & Environment Sciences, Chungbuk National University, Cheongju 28644, Republic of Korea; anjinpyo@chungbuk.ac.kr

**Keywords:** *Magnolia liliiflora*, denudatone, anti-neuroinflammation, BV2 microglial cells, lignans

## Abstract

**Background/Objectives**: Neuroinflammation is a key contributor to neurodegenerative diseases. *Magnolia liliiflora* Desr. is a traditional medicinal plant with therapeutic potential; however, its bioactive constituents and mechanisms remain unclear. This study aimed to identify active compounds from *M. liliiflora* leaves that inhibit inflammatory responses in microglial BV-2 cells. **Methods:** Anti-inflammatory activity was assessed by measuring nitric oxide (NO) production in lipopolysaccharide (LPS)-stimulated BV2 microglial cells. UPLC–qTOF MS/MS-based metabolite profiling combined with bioactivity-guided analysis was used to identify candidate biomarkers, which were subsequently isolated and structurally characterized. Network pharmacology and molecular docking analyses were performed to predict potential molecular targets and mechanisms of action. The effects on NF-κB signaling and inducible nitric oxide synthase (iNOS) and cyclooxygenase-2 (COX-2) expression were further validated by Western blot analysis. **Results**: Two previously undescribed lignans (**1** and **2**) and five known lignan derivatives (**3**–**7**) were isolated from the leaves of *M. liliiflora*. At 20 µM, compounds **1**, **3**–**5**, and **7** exhibited moderate inhibitory effects on nitric oxide (NO) production in lipopolysaccharide (LPS)-stimulated BV2 microglial cells, with 23%, 33%, 69%, 56% and 49% inhibition, respectively, and no detectable cytotoxicity. Notably, an ethyl acetate-derived enriched subfraction showed 97% inhibition of NO production at 10 µg/mL, suggesting potential synergistic activity of *M. liliiflora* lignans. Network pharmacology and molecular docking analyses predicted interactions between the isolated lignans and NF-κB pathway-related targets, thereby guiding subsequent experimental validation. Both compounds significantly reduced the expression of iNOS and COX-2 and suppressed LPS-induced activation of the NF-κB signaling pathway in a concentration-dependent manner, as confirmed by Western blot analysis. Overall, the results demonstrate that *M. liliiflora* leaves are a source of bioactive lignans that attenuate microglial activation by inhibiting NO production and key inflammatory mediators, effects that are associated with the suppression of the NF-κB signaling pathway. **Conclusions:** This study identified bioactive lignans from *M. liliiflora* leaves and demonstrated their anti-inflammatory activity in microglial cells. The findings establish the structural identities of the active compounds and confirm that *M. liliiflora* leaves are a valuable source of lignans with therapeutic potential for neuroinflammatory and neurodegenerative disorders.

## 1. Introduction

Neurodegenerative diseases (NDDs), including Alzheimer’s disease (AD), Parkinson’s disease (PD), cerebral ischemia, and multiple sclerosis, are major global health challenges strongly associated with aging and persistent neuroinflammation [1,2,3,4,5,6,7,8,9]. Although neuroinflammation initially plays a protective role by promoting brain tissue repair and clearance of cellular debris, its chronic and dysregulated activation leads to sustained release of pro-inflammatory mediators, including nitric oxide (NO), and increased oxidative stress. These processes contribute to neuronal dysfunction and exacerbate the progression of NDDs [10,11,12]. Microglial M1/M2 polarization is a hallmark of neuroinflammation, in which the tissue-repairing M2 phenotype shifts to the neurotoxic M1 phenotype, characterized by the production of pro-inflammatory cytokines (TNF-α, IL-1β, IL-6), reactive oxygen species, and nitric oxide through iNOS [13]. Therefore, inhibiting microglial activation has emerged as a promising therapeutic approach for the management of NDDs. Active and passive immunotherapeutic strategies [14], as well as small-molecule approaches targeting signaling pathways, including the NLRP3 inflammasome, Bruton’s tyrosine kinase, NF-κB, and NRF2, are under active investigation [15,16,17,18]. However, these pharmacological interventions have thus far yielded modest clinical benefits [19], emphasizing the need for alternative strategies and an expanded repertoire of bioactive molecules that can modulate microglia-mediated neuroinflammation.

Among emerging approaches, dietary phytochemicals have attracted scientific interest for their potential to alleviate chronic neuroinflammation, offering promising complementary approaches to prevent or slow the advancement of neurodegenerative diseases [20]. Widely studied phytochemicals include curcumin, resveratrol, and quercetin, whose beneficial effects have been linked to the modulation of NF-κB, the NLRP3 inflammasome, NRF2 signaling, and microbiome-mediated gut–brain communication [21,22,23]. While much of this work has focused on flavonoids, terpenoids, and alkaloids from well-characterized plant species, relatively underexplored botanical sources and plant tissues remain a rich reservoir of bioactive chemical diversity. Within this context, Magnolia species, long used in East Asian traditional medicine for the management of anxiety, depression, asthma, and inflammatory disorders, represent a particularly compelling phytochemical source of bioactive compounds [24,25,26,27]. The documented anti-inflammatory and neuroprotective activities of Magnolia species, together with evidence that the neolignans honokiol and magnolol modulate oxidative stress and inflammatory signaling in neuronal and glial systems [28,29], indicate that this genus harbors metabolites capable of influencing microglia-mediated neuroinflammation in neurodegenerative diseases.

Nevertheless, most phytochemical and pharmacological studies regarding anti-inflammatory activity have focused on the bark of *M. salicifolia, M. grandiflora, M. obovata,* and *M. officinalis*. In contrast, the phytochemistry and anti-inflammatory potential of *Magnolia liliiflora* remain comparatively underexplored, particularly in leaves. Although the flowers of *M. liliiflora* are commercially valued and widely consumed as herbal teas, the leaves have not yet been fully exploited as a source of bioactive metabolites [30]. Existing reports indicate that *M. liliiflora* leaves produce lignans, alkaloids, and phenolic acids, which likely reinforce its therapeutic potential [31,32]. On this basis, we hypothesized that leaf-derived extracts from *M. liliiflora* would yield bioactive-enriched preparations capable of modulating microglia-driven neuroinflammation by downregulating key inflammatory targets.

To test this hypothesis, we applied an untargeted, biomarker-guided UPLC-MS/MS strategy to prioritize candidate metabolites, correlating features from selected Magnolia extracts and fractions with nitric oxide (NO) inhibition in LPS-stimulated BV2 microglial cells. Guided by this analysis, we subsequently isolated, structurally characterized, and experimentally validated metabolites predicted to exhibit anti-neuroinflammatory activity from the ethanolic extract of *M. liliiflora* leaves. Furthermore, we aimed to examine the possible mechanism of action of the identified compounds using in silico docking and in vitro assays.

## 2. Materials and Methods

### 2.1. General Experimental Procedures

Optical rotations were recorded using a JASCO P-2000 polarimeter (JASCO International Co., Ltd., Tokyo, Japan). UV and electronic circular dichroism (ECD) spectra were measured with a Chirascan-Plus spectropolarimeter (Applied Photophysics Ltd., Surrey, UK). Infrared (IR) spectra were obtained using a Nicolet 6700 FT-IR spectrometer (Thermo Fisher Scientific, Waltham, MA, USA). 1D and 2D NMR spectra were recorded in deuterated solvents using a JNM-ECA 400 MHz spectrometer (JEOL Ltd., Tokyo, Japan). High-resolution electrospray ionization mass spectrometry (HRESIMS) was performed using a Waters Xevo G2 QTOF mass spectrometer (Waters MS Technologies, Manchester, UK) equipped with an electrospray ionization (ESI) source. For compound isolation, a Gilson HPLC purification system (Gilson, Villiers-le-Bel, France) was used with UV detection at 205 and 284 nm, employing an Optimapak C_18_ column (10 × 250 mm, 10 μm; Optimapak, Daejeon, Republic of Korea). Column chromatography (CC) was conducted using silica gel (63–200 μm; Zeochem AG, Rüti, Switzerland), RP-C18 (75 μm; Nacalai Tesque, Kyoto, Japan), and Sephadex LH-20 (GE Healthcare, Little Chalfont, UK). Medium-pressure liquid chromatography (MPLC) was performed using an Isolera™ system (Biotage, Cardiff, UK) with a Reveleris flash cartridge C18 column (120 g; Grace Reveleris, New Castle, DE, USA)). Industrial-grade solvents were used for extraction and purification, while analytical-grade acetonitrile (MeCN) and methanol (MeOH) were used for HPLC. All solvents were purchased from Daejung Chemical (Siheung, Republic of Korea).

### 2.2. Plant Materials

A total of 4.2 kg of *M. liliiflora* leaves were collected in October 2021 from the Herbarium of the Medicinal Plant Garden at the College of Pharmacy, Seoul National University (Goyang-si, Gyeonggi-do, Republic of Korea; 37°42′42″ N, 126°49′4″ E). A voucher specimen (No. 2021-SNU-09) was deposited in the Herbarium of the College of Pharmacy, Seoul National University, Seoul, Republic of Korea.

### 2.3. Preparation of Magnolia Leaves EtOAc Fractions for LC-MS/MS Analysis

Fifty milligrams of the EtOAc fraction of *M. liliiflora* was subjected to normal-phase chromatography using a stepwise solvent system of *n*-hexane/EtOAc/MeOH (*v*/*v*/*v*) with the following compositions: 10/0/0, 7.5/2.5/0, 5/5/0, 2.5/7.5/0, 0/10/0, and 0/7.5/2.5. This procedure yielded six subfractions corresponding to each step. Each subfraction was dissolved in HPLC-grade MeOH (1.0 mg/mL), filtered through a membrane filter (Advantec, Tokyo Roshi Kaisha, Tokyo, Japan), and a 2 μL aliquot was injected for LC-MS/MS analysis. For the EtOAc fractions of other Magnolia species, the same concentration and injection volume were used.

### 2.4. UPLC-qTOF-MS/MS Experiments

UPLC-MS/MS analysis was performed using an Agilent 6530 Q-TOF mass spectrometer coupled to an Agilent 1260 Infinity UHPLC system (Agilent Technologies, Santa Clara, CA, USA). Chromatographic separation was achieved on a Waters ACQUITY UHPLC^®^ BEH C18 column (100 mm × 2.1 mm, 1.7 μm; Waters, Co., Ltd., Manchester, UK) maintained at 40 °C. The system was operated in fast data-dependent acquisition (DDA) mode. The mobile phase consisted of water containing 0.1% formic acid (A) and acetonitrile containing 0.1% formic acid (B), with the following gradient: 10–90% B (0–20 min), 100% B (20.1–22.0 min), followed by re-equilibration to 10% B (22.1–24.0 min). Electrospray ionization (ESI) was operated in both positive and negative ion modes with an *m*/*z* scan range of 100 to 1500. Source parameters were as follows: sheath gas temperature, 350 °C; gas flow rate, 10 L/min; nebulizer pressure, 30 psi; capillary voltage (VCap), 4000 V; nozzle voltage, 1000 V; fragmentor voltage, 180 V; skimmer voltage, 65 V; octopole RF peak, 750 V; and collision energy, 50 eV.

### 2.5. Bioactivity Correlation with UPLC-MS/MS-Derived Features

Raw data from both ionization modes were converted to mzXML files using MSConvert in Proteowizard (v3.0.22317, ProteoWizard, Palo Alto, CA, USA) [33]. UPLC-MS/MS data from *M. liliiflora* fractions and selected *Magnolia* species were processed using MZMine 3 (v3, MZmine Development Team, Prague, Czech Republic) [34]. Positive-mode mzXML files were imported, and centroided spectra and positive ion mode were selected. MS1 and MS2 noise thresholds were set to 1.0 × 10^4^ and 1.0 × 10^2^, respectively. Chromatograms were built using a minimum of two consecutive scans, a minimum intensity of 1.0 × 10^4^, and a minimum height of 1.0 × 10^4^. Deconvolution was performed using the Local Minimum Resolver. Isotopes were grouped within a 10 ppm tolerance. Feature alignment was performed using the RANSAC aligner with 20 ppm *m*/*z* and 0.50 min retention-time tolerances, applying a linear model (threshold, 0.9). Gap filling and duplicate filtering were subsequently applied. The final feature list was exported as an mgf file and the quantification table as a csv file. The quantification table was formatted, and bioactivity data were incorporated. Data normalization (median), transformation (log10), and autoscaling were performed using MetaboAnalyst (v5.0, McGil University, Montreal, QC, Canada) [35]. Spearman rank correlation analysis was used to evaluate associations between feature intensities and NO inhibition, with *p*-values and false discovery rate (FDR) values calculated.

### 2.6. Feature-Based Molecular Networking

The final mgf file was uploaded to the GNPS platform for feature-based molecular networking [36]. A cosine score threshold of 0.65 and a minimum of four matched fragment ions were applied for network construction and spectral library searches. MS/MS-based annotation was performed using DEREPLICATOR [37]. The molecular networking job and associated parameters are available at https://gnps.ucsd.edu/ProteoSAFe/status.jsp?task=12df8aa68d72430390b53e46dedeefdf (accessed on 5 May 2025). The resulting molecular network was visualized using Cytoscape (v3.10, Cytoscape Consortium, San Diego, CA, USA) [38], and compound class annotation was carried out with MolNetEnhancer [39].

### 2.7. Extraction and Isolation

Air-dried *M. liliiflora* leaves were cut into small pieces and extracted three times with 70% EtOH by sonication at room temperature for 90 min each. The combined extracts were concentrated under reduced pressure, suspended in water, and successively partitioned with *n*-hexane, ethyl acetate (EtOAc), and n-butanol. The EtOAc fraction (150.3 g) was subjected to silica gel column chromatography (10 × 60 cm, 60–200 μm) using a gradient of *n*-hexane/EtOAc/MeOH (from 1/0/0 to 0/0/1, *v*/*v*/*v*), yielding 10 fractions (F1–F10). Fraction F8 (1.5 g) was further fractionated by MPLC using a MeOH/H_2_O gradient (60–80% MeOH) to afford subfractions F8.1–F8.7. Compound **5** (63.9 mg) was obtained from F8.2, and compound **1** (9.9 mg) was obtained from F8.5 by HPLC on an Optima Pak C18 column (4.6 × 250 mm, 5 μm particle size, RS Tech, Daejon, Republic of Korea) using isocratic MeCN/H_2_O (0.1% formic acid) at 45:65 (*v*/*v*) and a flow rate of 2 mL/min. Fraction F9 (0.5 g) was subjected to MPLC using a MeOH/H_2_O gradient (50–100% MeOH), yielding compounds **3** (59.6 mg) and **4** (69.8 mg). Fraction F7 (12.7 g) was purified by silica gel column chromatography (10 × 60 cm; 40–63 μm) using a gradient of *n*-hexane/EtOAc/MeOH (9/1/0 to 0/0/1), affording 11 subfractions (F7.1–F7.11). Subfraction F7.5 (5.7 g) was further processed by MPLC with MeOH/H_2_O (0.1% formic acid, 50–100% MeOH) to yield subfractions F7.5.1–7.5.7. Compound **7** (81.7 mg) was isolated from F7.5.2 by Sephadex LH-20 chromatography (100% MeOH). Crystals of compound **6** (81.4 mg) were obtained by cooling an oversaturated solution of F.7.5.5. The filtrate was further separated by Sephadex LH-20 (100% MeOH), affording five subfractions (F7.5.5.1–7.5.5.5). Finally, compound **2** (2.0 mg) was purified from F7.5.5.4 by HPLC using a MeCN/H_2_O gradient (0.1% formic acid, 45–65% MeCN).

### 2.8. Physicochemical Properties of Isolated Compounds ***1***–***7***

*Liliiflorin* F (**1**): White amorphous powder; ${\left[\alpha \right]}_{D}^{25}$ = 68.5 (*c* 0.5, MeOH); UV λmax (MeOH) (log ɛ) (nm) 206 (2.02), 233 (0.87), and 281 (0.24); ECD (MeOH) λ (Δε) 210 (14.9), 226 (−14.0), 252 (19.3), and 299 (−2.49) nm; IR (KBr) *ν*_max_ 2965, 2897, 1650, 1611, 1497, 1244, 1146, 1036, 839, 675 cm^−1^; ^1^H NMR (chloroform-*d*, 400 MHz) and ^13^C NMR (chloroform-*d*, 100 MHz): Table 1; HRESIMS: found *m*/*z* 403.2108 [M + H]^+^ (calcd for C_23_H_31_O_6_ at 403.2121, *m*/*z* error—1.3 ppm). (Figures S1–S11).

*Liliiflorin* G (**2**): White amorphous powder; ${\left[\alpha \right]}_{D}^{25}$ = 15.8 (*c* 0.5, MeOH); UV λmax (MeOH) (log ɛ) (nm) 208 (2.03), 231 (1.29), and 280 (0.56); ECD (MeOH) λ (Δε) 230 (−2.60), 246 (10.22), and 277 (4.56) nm; IR (KBr) *ν*_max_ 2955, 1648, 1606, 1509, 1459, 1133, 1009, 843, 677cm^−1^; ^1^H NMR (chloroform-*d*, 400 MHz) and ^13^C NMR (chloroform-*d*, 100 MHz): Table 1; HRESIMS: found *m*/*z* 373.1642 [M + H]^+^ (calcd for C_21_H_25_O_6_ at 373.1651, *m*/*z* error—2.4 ppm). (Figures S12–S21).

*Futoenone* (**3**): ^1^H NMR (400 MHz, chloroform-*d*) δ 6.73 (dd, *J* = 7.9, 1.1 Hz, 1H), 6.68 (t, *J* = 1.4 Hz, 1H), 6.64 (dd, *J* = 8.0, 1.4 Hz, 1H), 5.94 (s, 2H), 5.79 (s, 1H), 5.46 (s, 1H), 5.04 (t, *J* = 5.6 Hz, 1H), 3.66 (d, *J* = 1.1 Hz, 3H), 2.55 (td, *J* = 11.5, 6.2 Hz, 1H), 2.37 (ddd, *J* = 11.5, 6.5, 1.8 Hz, 1H), 2.28 (dt, *J* = 12.4, 5.4 Hz, 1H), 2.17 (d, *J* = 11.4 Hz, 1H), 2.02 (dq, *J* = 12.7, 6.5 Hz, 1H), 1.71 (dd, *J* = 14.1, 11.7 Hz, 1H), 0.59 (dd, *J* = 6.5, 1.0 Hz, 3H). ^13^C NMR (100 MHz, chloroform-*d*) δ 183.3, 180.2, 153.4, 148.0, 146.5, 137.4, 121.2, 109.1, 108.5, 107.8, 101.5, 101.1, 82.0, 55.4, 50.4, 46.2, 45.6, 43.7, 38.1, 14.5. (Figure S22) HRESIMS: found *m*/*z* 341.1386 [M + H]^+^ (calcd for C_20_H_21_O_5_ at 341.1389, *m*/*z* error—0.9 ppm).

*Denudatone* (**4**): ^1^H NMR (400 MHz, chloroform-*d*) δ 6.37 (s, 2H), 5.79 (s, 1H), 5.48 (s, 1H), 5.04 (t, *J* = 5.5 Hz, 1H), 3.84 (s, 6H), 3.81 (s, 3H), 3.66 (s, 3H), 2.55 (ddd, *J* = 11.5, 11.4, 6.1 Hz, 1H), 2.37 (dd, *J* = 11.3, 5.5 Hz, 1H), 2.29 (ddd, *J* = 14.3, 6.1, 5.4 Hz, 1H), 2.21 (d, *J* = 11.3 Hz, 1H), 2.06 (dd, *J* = 11.4, 6.5 Hz, 1H), 1.74 (dd, *J* = 14.0, 11.5 Hz, 1H), 0.60 (d, *J* = 6.5 Hz, 3H). ^13^C NMR (100 MHz, chloroform-*d*) δ 183.3, 180.3, 153.4, 139.2, 109.1, 104.7, 101.5, 82.0, 61.0, 56.3, 55.4, 50.5, 47.0, 45.4, 43.8, 37.9, 14.7. (Figure S23) HRESIMS: found *m*/*z* 387.1806 [M + H]^+^ (calcd for C_22_H_27_O_6_ at 387.1708, *m*/*z* error 0.2 ppm).

*cis-Burchelin* (**5**): ^1^H NMR (400 MHz, chloroform-*d*) δ 6.82 (d, *J* = 7.8 Hz, 1H), 6.71 (s, 2H), 5.98 (s, 2H), 5.92 (d, *J* = 5.1 Hz, 1H), 5.86 (d, *J* = 0.7 Hz, 1H), 5.80–5.68 (m, 2H), 5.50 (s, 1H), 5.21 (dd, *J* = 10.1, 1.7 Hz, 1H), 5.13 (dd, *J* = 16.9, 1.6 Hz, 1H), 3.68 (s, 4H), 3.19–3.13 (m, 1H), 2.74–2.64 (m, 2H), 2.53 (dd, *J* = 13.7, 6.7 Hz, 1H), 0.51 (d, *J* = 7.3 Hz, 3H). ^13^C NMR (100 MHz, chloroform-*d*) δ 182.9, 181.7, 153.0, 148.0, 147.5, 131.9, 130.6, 120.47, 119.0, 109.2, 108.4, 106.3, 102.1, 101.3, 87.4, 55.4, 54.1, 44.7, 44.0, 12.2. (Figure S24) HRESIMS: found *m*/*z* 341.1386 [M + H]^+^ (calcd for C_20_H_21_O_5_ at 341.1389, *m*/*z* error—0.9 ppm).

*(+)-Veraguensin* (**6**): ^1^H NMR (400 MHz, chloroform-*d*) δ 7.07 (d, *J* = 1.9 Hz, 1H), 7.04 (dd, *J* = 8.1, 1.9 Hz, 1H), 6.90–6.82 (m, 4H), 5.14 (d, *J* = 8.7 Hz, 1H), 4.42 (d, *J* = 9.3 Hz, 1H), 2.30–2.18 (m, 1H), 1.85–1.69 (m, 1H), 1.07 (d, *J* = 6.5 Hz, 3H), 0.66 (d, *J* = 7.0 Hz, 3H). ^13^C NMR (100 MHz, chloroform-*d*) δ 149.1, 148.7, 148.7, 148.2, 134.0, 133.6, 119.4, 118.8, 111.2, 110.8, 110.5, 110.1, 87.4, 83.2, 56.1, 56.0, 56.0, 55.9, 48.1, 46.1, 15.2, 15.1. (Figure S25) HRESIMS: found *m*/*z* 373.2024 [M + H]^+^ (calcd for C_22_H_29_O_5_ at 373.2015, *m*/*z* error 2.4 ppm).

*Nectandrin B* (**7**) ^1^H NMR (401 MHz, chloroform-*d*) δ 6.96 (d, *J* = 1.7 Hz, 2H), 6.97–6.88 (m, 4H), 4.50 (d, *J* = 6.6 Hz, 2H), 3.87 (s, 6H), 2.38–2.26 (m, 2H), 1.03 (d, *J* = 6.7 Hz, 6H). ^13^C NMR (100 MHz, chloroform-*d*) δ 146.6, 145.2, 134.3, 119.4, 114.2, 109.3, 87.5, 56.0, 44.4, 13.0. (Figure S26) HRESIMS: found *m*/*z* 345.1702 [M + H]^+^ (calcd for C_20_H_25_O_5_ at 345.1701, *m*/*z* error–0.3 ppm).

### 2.9. Computational Methods

Network Pharmacology. SMILES strings of the active compounds (**1** and **3**–**5**, **7**) were submitted to the Super-PRED webserver for target prediction. Super-PRED uses machine-learning models trained on 3D chemical structures and bioactivity data from the ChEMBL database [40]. Predicted protein targets were obtained with associated probabilities and model accuracies. Targets were ranked by the product of prediction probability and model accuracy, and only those with a combined score greater than 0.70 were retained. The combined target set for all compounds was used to construct a “Predicted Target” network in Cytoscape 3.10. Proteins associated with the Toll-like receptor, NF-kB, and NOD-like receptor signaling pathways were retrieved from the STRING database, imported into Cytoscape, and merged to form a unified reference network. Proteins common to both networks were identified and mapped to generate the final “Target Protein” network.

Molecular Docking. Crystal structures of selected targets were obtained from the RCSB Protein Data Bank (PDB), including NF-kB (p50; PDB ID: 1SVC) and an NF-kB complex (PDB ID: 1NFK), for molecular docking studies performed in Discovery Studio (BIOVIA, Dassault Systems). Ligands were energy-minimized, and proteins were prepared by assigning ionization states at pH 7.5. Binding sites were selected based on PDB annotations, and docking was performed using the CDOCKER protocol. Docking was computationally validated based on available ligands for each protein structure.

Pharmacokinetic Parameter Prediction. SMILES strings for each compound were used as input in the SwissADME website [41].

### 2.10. Cell Culture

BV2 microglial cells were obtained from the American Type Culture Collection (ATCC, Manassas, VA, USA). Cells were maintained in Dulbecco’s Modified Eagle’s Medium (DMEM; Welgene, Republic of Korea) supplemented with 10% fetal bovine serum (FBS; Gibco, Waltham, MA, USA) and 1% penicillin/streptomycin (Gibco). Cultures were incubated at 37 °C in a humidified atmosphere containing 5% CO_2_.

### 2.11. LPS-Induced NO Production and Cell Viability

Nitric oxide (NO) production was measured using the Griess assay, which quantifies nitrite as a stable NO metabolite. BV2 cells were seeded into 96-well plates at 1 × 10^4^ cells/well and incubated overnight. Cells were then co-treated with LPS (1 μg/mL) and the extract (10 μg/mL), fractions (10 μg/mL) or isolated compounds (20 μM). Curcumin (10 μM) was employed as a positive control, and a negative control (in the absence of test compounds) was also included. Test compounds or fractions were prepared in serum-free medium from DMSO stock solutions. Treated cells were incubated for an additional 24 h. An equal volume of solution A (1% sulfanilamide) and solution B (5% phosphoric acid containing 0.1% naphthyl ethylenediamine dihydrochloride) was added to culture supernatants, and absorbance was measured at 540 nm using a microplate reader (VersaMaxTM, Molecular Devices, San Jose, CA, USA). Nitrite concentrations were determined from a sodium nitrite standard curve. Percentage inhibition was calculated using the following formula: % inhibition = 1 − (NO_treatment_/NO_LPS_). Cell viability was assessed using the 3-(4,5-dimethyl-2-thiazolyl)-2,5-diphenyl-2H-tetrazolium bromide (MTT) assay. Cells were treated and incubated in the NO production test likewise. Subsequently, 20 µL of MTT solution (2 mg/mL) was added to each well and incubated for 4 h in the dark. After removing the supernatant, formazan crystals were dissolved in DMSO, and absorbance was measured at 570 nm. All experiments were performed in triplicate.

### 2.12. Western Blot Analysis

BV2 cells were seeded into 6-well plates at 2 × 10^5^ cells/well and incubated overnight. Cells were pretreated with the isolated compounds for 1 h and then stimulated with LPS (1 μg/mL) for 24 h. Dexamethasone (20 µM) was used as positive control. Cells were lysed in RIPA buffer (Bio-Rad, Hercules, CA, USA) supplemented with protease and phosphatase inhibitor cocktails (Roche Diagnostics GmbH, Manheim, Germany). Lysates were centrifuged at 12,000 rpm for 15 min at 4 °C, and supernatants were collected. Protein concentrations were determined using a BSA assay. Equal amounts of protein were mixed with SDS-PAGE loading buffer (GeneAll Biotechnology Co., Ltd., Seoul, Republic of Korea), boiled for 10 min, separated on 8% SDS-PAGE gels, and transferred to PVDF membranes. After blocking, membranes were incubated overnight at 4 °C with primary antibodies against COX-2 (1:2000, Abfrontier, Seoul, Republic of Korea) and iNOS (1:2000, Thermo Fischer Scientific, Waltham, MA, USA). Membranes were washed with TBST and incubated with HRP-conjugated secondary antibodies, including goat anti-rabbit IgG-HRP (Invitrogen, Carlsbad, CA, USA) and goat anti-mouse IgG-HRP (Gen-DEPOT, Katy, TX, USA), for 1 h at room temperature. Bands were visualized using an enhanced chemiluminescence (ECL) kit (Abfrontier). Band intensities were quantified in ImageJ (v1.54p) and normalized to β-actin. Experiments were performed in triplicate.

### 2.13. Statistical Analyses

Biological experiments involving group comparisons were analyzed using one-way analysis of variance (ANOVA) to determine significant differences among groups, followed by Tukey’s post hoc test. A *p*-value of < 0.05 was considered statistically significant (* *p* < 0.05, ** *p* < 0.01, *** *p* < 0.001 and **** *p* < 0.0001) as determined using GraphPad Prism (v10.2.1, GradphPad Software Inc., San Diego, CA, USA) Spearman rank correlation analysis was conducted to evaluate the association between metabolite feature intensities and nitric oxide (NO) inhibition responses using the MetaboAnalyst platform [35]. For each metabolite feature, a nonparametric Spearman correlation coefficient was calculated with the corresponding NO inhibition values, and the significance of the association was assessed using two-sided *p*-values. To account for multiple testing across all features, *p*-values were adjusted using the false discovery rate (FDR) approach, and FDR-adjusted q-values were used to identify statistically significant correlations.

## 3. Results

### 3.1. NO Inhibitory Properties of M. liliiflora and Identification of Bioactivity-Associated Metabolites

The anti-inflammatory potential of *M. liliiflora* was first assessed by its ability to inhibit LPS-induced nitric oxide (NO) production in BV2 microglial cells (Figure S27). Among the solvent-partitioned fractions of the 70% EtOH leaf extract, the EtOAc and BuOH fractions significantly suppressed NO production (Table S1), indicating enrichment of anti-inflammatory constituents in these medium-polarity fractions. We then applied an untargeted, biomarker-guided UPLC-qTOF-MS/MS analysis to EtOAc subfractions of *M. liliiflora* and EtOAc fractions (Table S2) from metabolically related *Magnolia* species (Table S3) to link chemical features with NO-inhibitory effects (Table S4). These related *Magnolia* extracts were selected using an in-house LC–MS/MS metabolite database constructed from *Magnolia* extracts (Figure S28), thereby providing a broader phytochemical background and reducing the likelihood of false-positive feature–activity associations. Linking metabolite features with bioactivity across these samples revealed a subset of ions that were positively correlated with NO inhibition. In other words, increased bioactivity across samples was associated with increased metabolite ion intensities. While *p*-values provided statistical significance, the false discovery rate (FDR) was additionally applied to reduce the likelihood of false positives. These analyses, summarized in Table 2, allowed the identification of candidate metabolites strongly correlated with the observed anti-inflammatory activity of *M. liliiflora*, with significance confirmed by FDR-adjusted values (FDR < 0.05; Table 2).

### 3.2. Chemical Profiling and Feature-Based Molecular Networking

**Figure 1:**
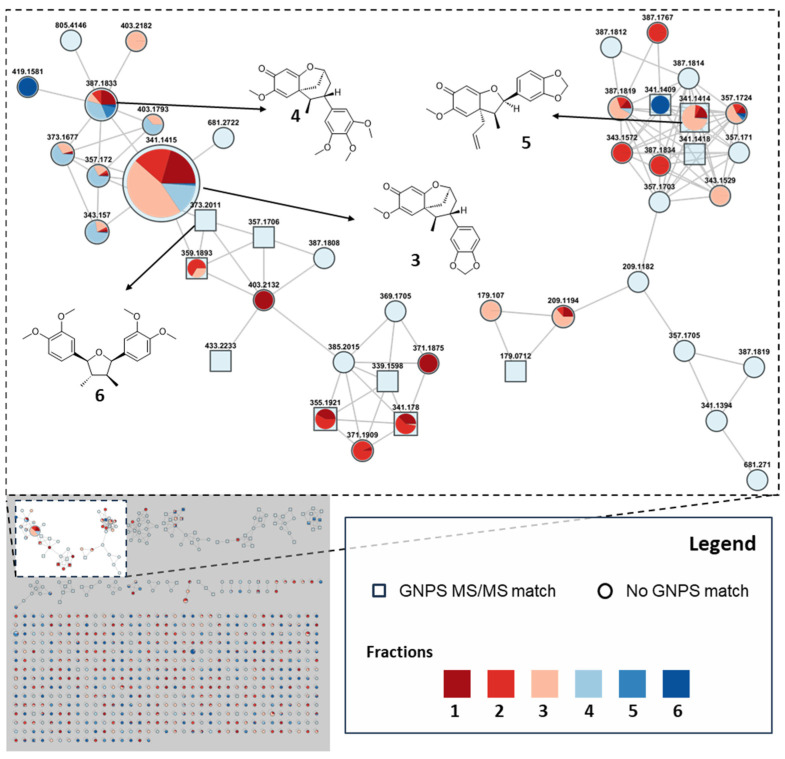
Feature-based molecular networking (FBMN) analysis of the EtOAc fraction of *Magnolia liliiflora*. The molecular network reveals major clusters enriched in lignans, including furanoid, furofuranoid, arylnaphthalene, and neolignan subclasses, as well as a distinct alkaloid cluster (Figure S30). Candidate bioactivity-associated features identified by correlation analysis are highlighted. The chemical structures of the isolated compounds **3**–**6** are shown.

The total ion chromatogram (TIC) and base peak chromatogram (BPC) acquired in positive ionization mode revealed three major constituents in the EtOAc fraction: *m*/*z* 387.1833 [M + H]^+^ at 8.28 min, *m*/*z* 341.1415 [M + H]^+^ at 9.27 min, and *m*/*z* 341.1413 [M + H]^+^ at 10.27 min (Figure S29). Notably, the first two features were also identified as candidate biomarkers in the correlation analysis, supporting the notion that these bioactive constituents are present at appreciable levels in the extract. To further investigate the chemical identities of these features, feature-based molecular networking (FBMN) was applied to the UPLC-MS/MS dataset. This approach enabled the clustering of molecular features based on similarities in their MS/MS fragmentation patterns, thereby highlighting chemical similarities. It also facilitated compound annotation through comparison with the Global Natural Products Social Molecular Networking (GNPS) database (Table S5) [36]. The resulting molecular network (Figure 1) revealed two prominent clusters enriched in annotated lignans, including compounds belonging to furanoid, furofuranoid, arylnaphthalene, and neolignan classes (Figure S30, Table S5). These findings are consistent with previously reported lignan constituents of *M. liliiflora* [30,31]. In addition, a distinct molecular cluster corresponding to alkaloids was observed (Figure S31), which included the biomarker candidate *m*/*z* 266.1192 [M + H]^+^ at 5.41 min. This compound was annotated as anonaine, an isoquinoline alkaloid previously isolated from *Liriodendron chinensis* (Magnoliaceae) leaves and reported to exert anti-inflammatory effects by suppressing NO production in rat peritoneal macrophages [42]. In contrast, several of the most abundant features in the EtOAc extract, including the top bioactivity-associated biomarkers identified through correlation analysis, could not be annotated using available MS/MS spectral libraries. These unidentified features were therefore prioritized for targeted isolation and structural elucidation.

### 3.3. Structural Elucidation of Isolated Compounds from Magnolia liliiflora Leaves

**Figure 2:**
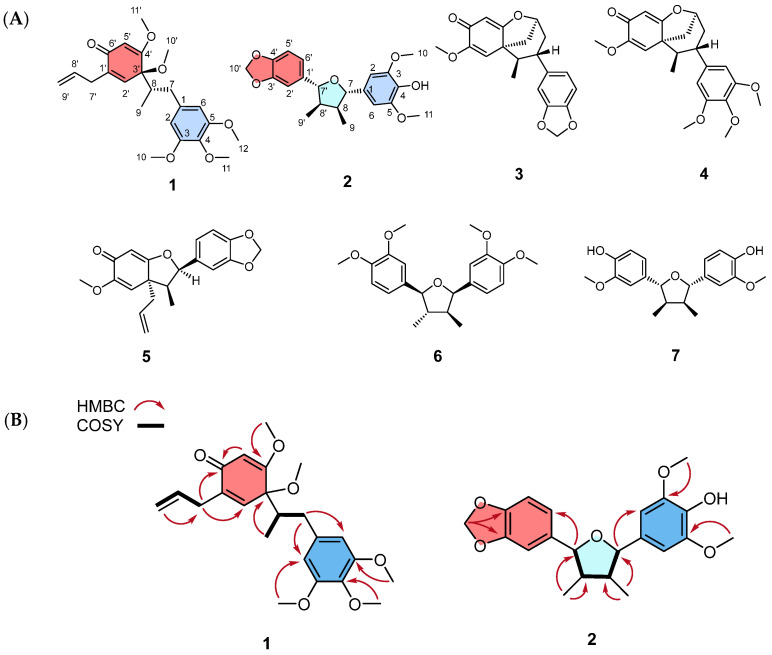
(**A**) Chemical structures of compounds **1─7** isolated from *M. liliiflora*. (**B**) Key COSY correlations (bold lines) and HMBC correlations (red arrows) observed for the new compounds **1** and **2**.

The 70% EtOH extract of the dried leaves of *M. liliiflora* was subjected to liquid–liquid partitioning, followed by chromatographic separation of the EtOAc fraction. This process led to the isolation of two highly probable biomarker candidates, which were identified as futoenone (**3**) and denudatone (**4**) (Figure 2, Table S6) based on comparisons with previously reported spectroscopic data [43]. In addition, two previously undescribed lignans (**1** and **2**) were isolated and structurally characterized, along with three known compounds: *cis*-burchelin (**5**), (+)-veraguensin (**6**), and nectandrin B (**7**) (Figure 2). Their structures were confirmed by comparison with published reference data [43,44,45,46,47,48]. The isolated compounds could be classified into two groups: lignans featuring a cyclohexanone core (compounds **1** and **3**–**5**) and those characterized by a substituted tetrahydrofuran skeleton (compounds **2**, **6**, and **7**). Among the former, futoenone (**3**) and denudatone (**4**) possess a spirocyclohexadienone moiety and represent the only two known spiro-(5,5)-undecanoids neolignans reported within the *Magnoliaceae* family [32]. The structures of the two newly identified compounds were elucidated using comprehensive spectroscopic analyses, further supported by quantum chemical calculations of their electronic circular dichroism (ECD) spectra.

**Figure 3:**
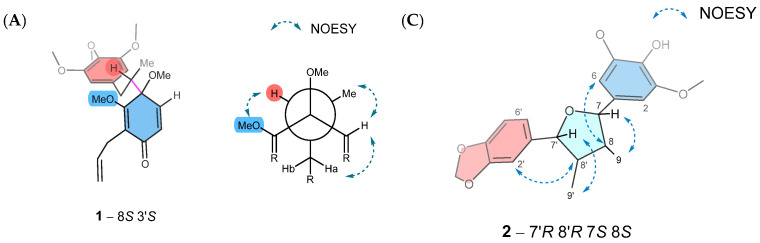

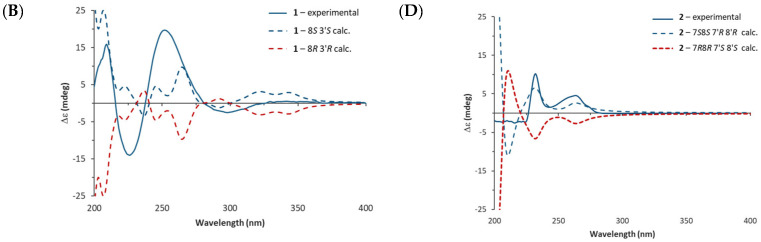
(**A**) Key NOESY correlations of compound **1**. (**B**) Calculated ECD spectrum of compound **1**. (**C**) *J*-coupling analysis for the relative configuration of compound **2.** (**D**) Calculated ECD spectrum of compound **2**.

Compound **1** was obtained as a white powder, and its molecular formula was determined to be C_23_H_30_O_6_ based on the HRESIMS ion peak at *m*/*z* 403.2110 [M + H]^+^ (calcd for C_23_H_31_O_6_, 403.2121). The ^1^H NMR spectrum displayed meta-coupled aromatic protons at δ_H_ 6.38 (s, 2H), two singlets attributable to olefinic protons at δ_H_ 6.33 (s, 1H) and 5.74 (s,1H), allylic protons at δ_H_ 5.16 (d, *J* = 5.6 Hz, 1H) and 5.13 (br s, 1H), and an additional olefinic proton at δ_H_ 5.90 (m, 1H). Five methoxy groups were observed at δ_H_ 3.85 (s, 6H), 3.82 (s, 3H), 3.78 (s, 3H), and 3.18 (s, 3H), along with a methyl group at δ_H_ 0.60 (d, *J* = 6.8 Hz, 3H). The ^13^C NMR spectrum exhibited 23 carbon signals, including one carbonyl carbon, twelve aromatic carbons, six oxygenated carbons, and four aliphatic carbons (Table 2). The connectivity between one aromatic ring and the C-7 to C-9 side chain was established by COSY correlations among protons H-7, H-8, and H-9, together with HMBC correlations from H-7 to C-2 and C-6, defining a phenylpropanoid subunit. Three methoxy groups attached to this aromatic ring were confirmed by HMBC correlations from methoxy protons to C-3, C-4, and C-5, respectively. A second phenylpropanoid subunit was identified through COSY correlations involving the allyl moiety and the H-7′ methylene protons, as well as HMBC correlations involving the allyl moiety and the H-7′ to C-1′, C-2′ and C-6′, from H-2′ to C-4′ and C-6′, and from H-5′ to C-3′ and C-6′ (Figure 2B). The linkage between the two phenylpropanoid units was supported by an HMBC correlation from H-9 to C-3′ (Figure 2B). Comparison with reported spectroscopic data for lancifolin C [49] indicated that compound **1** possesses an additional methoxy group at C-5. Furthermore, analysis of NOESY correlations in combination with calculated ECD spectra (Figure 3A,B) revealed that the stereochemistry of compound **1** differs from that of liliflorin E (8*R*,3′*S*) [30]. Accordingly, compound **1** was identified as a new lignan and named liliflorin F.

Compound **2** was also isolated as a white powder. Its molecular formula was assigned as C_21_H_24_O_6_ based on the HRESIMS ion peak at *m*/*z* 373.1642 [M + H]^+^ (calcd for C_21_H_25_O_6_, 373.1651). The ^1^H NMR spectrum revealed three aromatic protons at δ_H_ 6.97 (s, 1H), 6.87 (dd, *J* = 7.9, 1.7 Hz, 1H), and 6.78 (d, *J* = 7.9, 1H), consistent with an ABX spin system. In addition, meta-coupled aromatic protons appeared at δ_H_ 6.65 (s, 2H), along with a chemically equivalent methylene signal at δ_H_ 5.94 (s, 2H). Other characteristic signals included oxygenated protons at δ_H_ 5.46 (s, 1H) and 4.45 (dd, *J* = 6.6, 2.1 Hz, 2H), two methoxy groups at δ_H_ 3.90 (s, 6H), and two methyl groups at δ_H_ 1.03 (d, *J* = 6.6, 3H) and 1.00 (d, *J* = 6.6, 3H). A multiplet at δ_H_ 2.30 (m, 2H) was also observed. The ^13^C NMR spectrum showed 21 carbon resonances, including twelve aromatic carbons, four oxygenated carbons, and four aliphatic carbons (Table 2). An additional methylene carbon was identified through HSQC correlations between H-10 and C-10. HMBC correlations from H-10 to C-3 and C-4 supported the presence of a benzodioxolane moiety (Figure 2B). A second substituted aromatic ring was inferred from HMBC correlations of H-2 and H-6 with C-3, C-4, and C-5, as well as from the methoxy protons at δ_H_ 3.90 to C-3 and C-5. The symmetry of this aromatic ring was supported by the equivalent chemical shifts in H-2 and H-4, the methoxy signals, and a chelated hydroxyl proton at δ_H_ 5.46. Only two HMBC correlations were observed for methyl groups (Me-9 and Me-9′), suggesting their spatial proximity and supporting the presence of a tetrahydrofuran ring linking the benzodioxolane moiety and the second aromatic unit. This structural feature was further corroborated by the ten degrees of unsaturation calculated from the molecular formula. The planar structure of compound **2** closely resembled that of machilin G [50,51,52], except for an additional methoxy group at C-5. Comparison with reported data for related tetrahydrofuran-type lignans [53] suggested a *trans* configuration between H-7 and H-8, as well as between H-7′ and H-8′. The absolute configuration of compound **2** was further established by NOESY analysis and comparison with calculated ECD spectra (Figure 3C,D). Consequently, compound **2** was characterized as a new lignan and named liliflorin G.

### 3.4. Anti-Neuroinflammatory Effects of Isolated Compounds in LPS-Stimulated Microglia Cells

**Figure 4:**
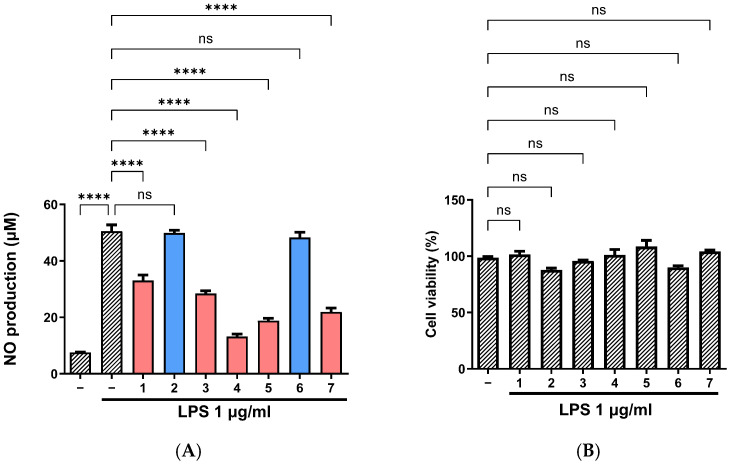
NO production inhibitory effects of isolated compounds on LPS-induced NO production and cell viability. (**A**) NO production (μM), (**B**) Cell viability (%) in BV2 microglial cells. BV2 cells were seeded at a density of 1 × 10^4^ cells/well in 96-well plates and incubated overnight to allow cell attachment. Cells were then stimulated with LPS (1 μg/mL) in the presence or absence of the isolated compounds (20 µM). Nitrite accumulation in the culture supernatant was quantified using the Griess reagent as an indicator of NO production. Cell viability was evaluated using the MTT assay. Data are expressed as the mean ± standard error of the mean (SEM) from three independent experiments. **** *p* < 0.0001 vs. control group; and **** *p* < 0.0001 vs. LPS-treated group; ns, not significant.

In consideration of the NO-inhibitory activity observed in the 70% EtOH extract and subfractions of *M. liliiflora* leaves, all isolated compounds were further evaluated for their ability to suppress NO production in LPS-stimulated BV2 microglial cells, a well-established in vitro model of neuroinflammation. At a concentration of 20 μM, compounds **1**, **3**–**5**, and **7** significantly reduced LPS-induced NO production compared with the LPS-treated control group (*p* < 0.0001), corresponding to inhibition rates of 23%, 33%, 69%, 56%, and 49%, respectively, as shown in Figure 4A. These results indicate that multiple constituents contribute to the extract’s NO-inhibitory activity. Among single compounds, compounds **4** (denudatone) and **5** (*cis*-burchelin) exhibited relatively strong inhibitory effects, suggesting that they may play a central role in the observed bioactivity. To verify that the suppression of NO production was not attributable to nonspecific cytotoxic effects, cell viability was assessed using an MTT assay following compound treatment. As illustrated in Figure 4B, none of the isolated compounds (**1**–**7**) caused a significant reduction in BV2 microglial cell viability at the tested concentration. Cell viability remained comparable to that of the untreated control group, thereby excluding cytotoxicity as a confounding factor and confirming that the observed NO inhibition reflects genuine anti-inflammatory activity.

### 3.5. Network Pharmacology and Plausible Mechanism of Action

**Figure 5:**
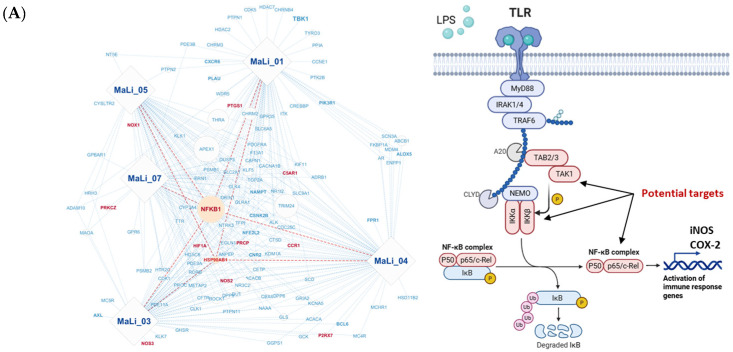

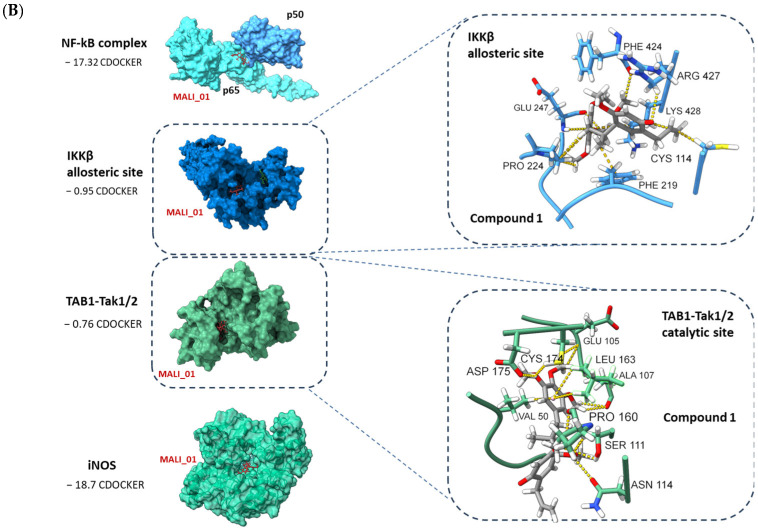
Network pharmacology analysis and identification of probable molecular targets of the isolated active compounds. (**A**) **Left**: Predicted target interaction network of the isolated active compounds, in which proteins associated with inflammatory pathways are highlighted in red. **Right**: Proposed mechanism by which the isolated lignans suppress the inflammatory response. (**B**) Molecular docking analysis suggests that IKKβ and the TAB1-TAK1/2 complex are putative molecular targets of the isolated compounds.

Although numerous lignans have been reported to exhibit anti-inflammatory activity, their precise molecular targets remain largely undefined [54]. Notably, lignans bearing a spirocyclohexadienone scaffold have not previously been reported to possess anti-inflammatory properties. To explore the potential mechanisms of action of the isolated lignans, their SMILES codes were submitted to the Super-PRED webserver [40] to predict putative biological targets. Based on these predictions, a compound–target interaction network was constructed. Targets with the highest probability scores were further analyzed using the STRING database and gene ontology (GO) enrichment analysis revealed significant associations with inflammatory-related pathways (Figure S32). Integration of these datasets led to the construction of a predictive target network (Figure 5A and Figure S33), which highlighted the NF-κB signaling pathway as the most likely route mediator of the observed bioactivity. Because these target predictions are derived from phenotypic data curated in the ChEMBL database [40], the proposed targets should be regarded as hypothetical. Nevertheless, based on current understanding of the NF-κB signaling cascade, particularly its activation through LPS-induced stimulation of Toll-like receptor 4 (TLR4) [36], several plausible molecular targets were proposed (Figure 5A). These include the TAB1/TAK1 complex, IKKβ, and the NF-κB p50/p65 heterodimer, all of which play central roles in the regulation of inflammation. To further investigate these hypotheses, molecular docking studies were performed. The molecular modelling results (Figure 5B and Figure S34) indicated that nectandrin B (**7**) exhibited the strongest overall binding affinities among the tested compounds (Table S7). In addition, compounds containing a cyclohexadienone moiety (**1** and **3**–**5**) showed favorable binding interactions with both the allosteric site of IKKβ and the catalytic domain of the TAB1-TAK1/TAK2 complex, suggesting potential multitarget activity (Table S7). While these in silico findings provide a rational framework for the proposed targets and mechanisms of action, further experimental validation is required to confirm these molecular interactions and their relevance in cellular systems.

### 3.6. Effect on iNOS and COX-2 Expression in LPS-Stimulated BV2 Microglial Cells

**Figure 6:**
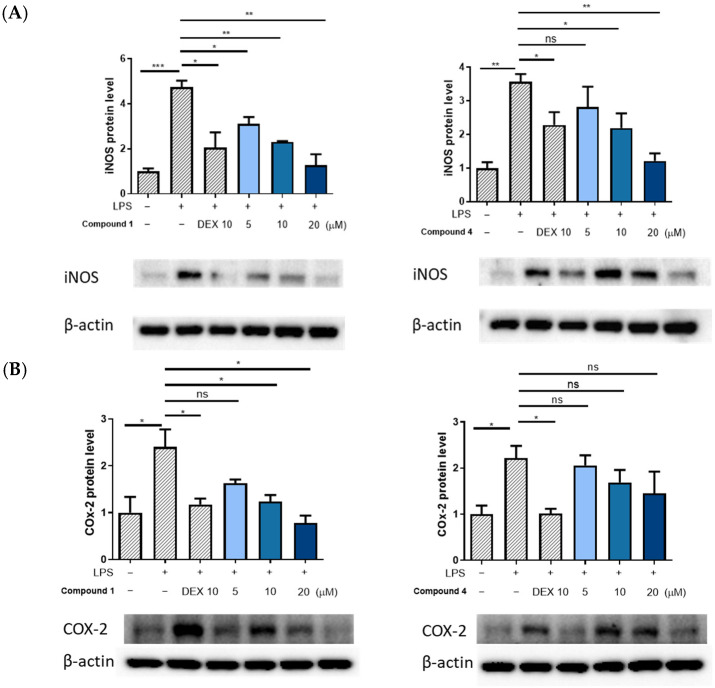
Effects of compounds **1** and **4** on iNOS and COX-2 expression in BV2 microglial cells. (**A**) Effects of compounds **1** and **4** on iNOS protein expression. BV2 cells were pretreated with the indicated concentrations of the compounds in 6-well plates for 1 h, followed by stimulation with LPS (1 μg/mL) for 24 h. iNOS protein levels were analyzed by Western blotting to assess concentration-dependent effects. Representative blots from three independent experiments are shown. (**B**) Effects of compounds **1** and **4** on LPS-induced COX-2 protein expression in BV2 microglial cells. Data are expressed as the mean ± standard error of the mean (SEM) from three independent experiments. * *p* < 0.05, ** *p* < 0.01, *** *p* < 0.001; ns, not significant.

Computational docking studies suggested that the isolated compounds exert regulatory effects on the NF-κB signaling pathway by targeting upstream components involved in the activation cascade preceding NF-κB-mediated transcription of pro-inflammatory genes. NF-κB is known to regulate the expression of iNOS and COX-2 by binding to the promoter regions of their respective genes, thereby inducing transcription in response to inflammatory stimuli [55]. Compound **1** and denudatone (**4**) were selected for further investigation as representative bioactive constituents. Although nectandrin B (**7**) also exhibited notable anti-inflammatory activity, it was not examined further in this study because its anti-inflammatory properties have already been well documented [56,57]. Considering both their pronounced anti-inflammatory activity and their relatively higher abundance in the plant extract, the previously unreported compound **1** and denudatone (**4**) were selected for further investigation of their effects on the NF-κB signaling pathway. Accordingly, the protein expression levels of iNOS and COX-2 were assessed in LPS-stimulated BV2 microglial cells. Western blot analysis demonstrated that both compounds reduced iNOS expression in a dose-dependent manner (Figure 6A). At a concentration of 20 μM, compound **1** and denudatone (**4**) suppressed iNOS protein levels by approximately 70%, an effect comparable to that observed with the positive control dexamethasone (10 μM) (*p* < 0.01, *t*-test). These results support the proposed mechanism by which these compounds exert anti-inflammatory effects through modulation of NF-κB signaling. Consistent with the iNOS results, compound **1** also reduced COX-2 protein expression in a dose-dependent manner, although to a slightly lesser extent. At 20 μM, compound **1** reduced COX-2 levels to an extent comparable to that of dexamethasone (10 μM), indicating a potent anti-inflammatory effect through dual inhibition of key inflammatory mediators. In contrast, denudatone (**4**) did not produce a statistically significant reduction in COX-2 expression, although a downward trend was observed (Figure 6B). Overall, both compounds exhibited significant anti-inflammatory activity in LPS-stimulated microglial cells. While these findings highlight their therapeutic potential, further studies are required to fully elucidate their molecular mechanisms of action. Nonetheless, the observed downregulation of iNOS and COX-2 strongly supports the notion that these lignans exert their anti-inflammatory effects, at least in part, through modulation of the NF-κB signaling pathway.

Overall, both compounds exhibited significant anti-inflammatory activity in LPS-stimulated microglial cells. While these findings highlight their therapeutic potential, additional studies are required to fully elucidate their molecular mechanisms of action. Nonetheless, the observed downregulation of iNOS and COX-2 strongly suggests that these lignans exert their effects, at least in part, through modulation of the NF-κB pathway.

### 3.7. In Silico Prediction of the Pharmacokinetic Properties of Isolated Compounds

To propose dietary phytochemicals that may effectively act as anti-neuroinflammatory materials, it is crucial that these molecules can cross the blood–brain barrier and reach their target sites within the brain. To this end, we next examined their predicted pharmacokinetic behavior using in silico ADME tools. Pharmacokinetic parameters were estimated with the SwissADME platform, which applies validated cheminformatic models to predict oral absorption, distribution, metabolism, and excretion from chemical structure. These calculations, which included parameters relevant to oral absorption, central nervous system penetration, and active efflux from the brain to the bloodstream (P-glycoprotein (P-gp) substrate status), are summarized in Table 3. In general, we observed that all compounds are predicted to be absorbed through the gastrointestinal tract and to be permeable across the blood–brain barrier (Table 3, Figure S35). Only nectandrin B was predicted to be a P-gp substrate and therefore actively transported out of the brain. These predictions additionally highlight the potential of *M. liliiflora* lignans to be further explored as dietary phytochemicals that may mitigate neuroinflammation.

## 4. Discussion

The well-documented challenge of limited reproducibility in the biological activity of ethnobotanical materials continues to hinder their clinical translation. In this study, we aimed to build a robust foundation for future investigations of *M. liliiflora* as a dietary source of inflammation-modulating phytochemicals. Our study addresses a knowledge gap regarding the biological relevance of the reported beneficial effects of *M. liliiflora* by providing a comprehensive metabolite profile and the clear identification of key bioactivity contributors. These findings provide valuable information to ensure the reproducibility of future pharmacological studies and support the advancement of translational research in both preclinical and clinical settings.

We investigated the anti-inflammatory properties of constituents from the 70% EtOH extract of *M. liliiflora* leaves, integrating UPLC-MS/MS-based profiling of EtOAc subfractions with quantitative assessment of nitric oxide (NO) production in LPS-stimulated BV2 microglial cells. Our novel approach included not only chemometric analysis of *M. liliiflora* subfractions but also the analysis of ethyl acetate extracts from metabolically related *Magnolia* species. Based on untargeted LC-MS fingerprints, a preliminary chemometric analysis of an in-house *Magnolia* extract library identified species that were metabolically similar to *M. liliiflora*, yet distinct from well-characterized *Magnolia* species. Given that the analysis of subfractions alone is susceptible to false-positive signals arising from co-eluting inactive constituents that may be carried forward as apparent “active” markers, we integrated metabolically similar extracts into the analysis to mitigate the co-elution-related bias and thereby strengthen the robustness of the inferred relationships between phytochemical patterns and bioactivity. Consequently, we systematically tracked the relationship between relative metabolite abundance and bioactivity by performing correlation analyses between LC-MS/MS-derived feature intensities and NO inhibitory activity. Our research strategy led to the identification of a subset of candidate bioactive chemical markers, including the known anti-inflammatory alkaloid, anonaine, as well as two isolated spirocyclohexadienone-type lignans, which were major constituents of the EtOAc fraction and showed significant inhibition of LPS-induced NO production in BV2 microglial cells.

Although lignans from various Magnolia species, including magnolol, honokiol, and structurally diverse neolignans, tetrahydrofurans, furofuranoids, and arylnaphthalenes, have been widely studied, the cyclohexadienone-type lignans identified here remain relatively underexplored. Our results indicate that these lignans are important contributors to the anti-inflammatory activity of the EtOAc fraction of *M. liliiflora* and represent the first description of inflammation-modulating activity for lignans bearing a cyclohexadienone scaffold. Notably, the newly identified cyclohexadienone-type lignan, compound **1**, also displayed modest inhibitory effects, further expanding the range of bioactive lignans associated with *M. liliiflora*.

Previous work has shown that lignans can reduce inflammatory responses by inhibiting NF-κB signaling, suppressing COX-2 expression, and reducing prostaglandin E2 (PGE2) production [58,59]. In line with this, our in silico network pharmacology and molecular docking analyses suggested that NF-κB signaling is a primary target of the isolated compounds. This hypothesis is supported by the observed reduction in iNOS and COX-2 expression in LPS-stimulated BV2 microglial cells treated with compound **1** and denudatone (**4**), which is consistent with previous reports describing lignans that suppress LPS-induced BV-2 microglial activation through inhibition of NF-κB signaling [60,61]. iNOS and COX-2 are well-known transcriptional targets within the NF-κB and MAPK pathways in LPS-activated microglia, suggesting that their downregulation reflects NF-κB modulation [62,63,64,65]. Importantly, selective COX-2 inhibition reduces neuroinflammatory signaling and ameliorates behavioral outcomes in animal models of neuroinflammation, as demonstrated by decreased prostaglandin E_2_ accumulation, reduced neutrophil infiltration, and attenuation of blood–brain barrier disruption following inflammatory challenge [66,67,68,69]. Likewise, genetic or pharmacological inhibition of iNOS mitigates inflammatory brain injury by suppressing toxic nitric oxide production and downstream oxidative stress [70,71]. Therefore, iNOS and COX-2 are more than mere inflammatory markers; they are important contributors to neuroinflammatory pathogenesis across multiple experimental models. On this basis, compounds that robustly and selectively downregulate iNOS and COX-2 in LPS-activated microglia can be reasonably proposed as candidate anti-neuroinflammatory agents.

In addition, in silico ADME predictions suggested that these compounds may be capable of crossing the blood–brain barrier and, in most cases, were predicted not to be substrates of major efflux pumps, consistent with experimental findings for structurally related lignans in cell-based in vitro models [72]. This information suggests that, in theory, these compounds may reach the brain and act on central inflammatory targets.

This study is limited by its reliance on in silico and in vitro assays in BV2 microglial cells using a restricted set of inflammatory readouts (NO production and COX-2 and iNOS expression), which, although informative, do not fully confirm the identified compounds as effective anti-neuroinflammatory agents. Future work will need to focus on broader mechanistic and in vivo validation, including additional in vitro models, animal studies, and pharmacokinetic profiling in models of inflammation-associated neuropathology.

## 5. Conclusions

This study contributes to advancing the research on dietary phytochemicals with potential anti-neuroinflammatory activity. Our study addresses a critical gap in understanding the pharmacological relevance of the reputed benefits of *M. liliiflora* by delivering a high-resolution metabolite profile together with unambiguous identification of the principal bioactive constituents. This integrated strategy, which combines detailed metabolite characterization with statistically validated attribution of bioactivity, represents an innovative approach for deconvoluting complex natural extracts and substantiating traditional claims with mechanistic evidence. Fraction-enriched lignans from *M. liliiflora*, and specifically in compound **1**, a newly identified cyclohexadienone-type lignan, and denudatone (**4**), exhibited inhibitory activities of LPS-induced NO production, as well as the expression of iNOS and COX-2, in BV2 microglial cells, implying that their effects are likely mediated, at least in part, through modulation of the NF-κB signaling pathway.

Collectively, these findings provide scientific support for the traditional medicinal use of *M. liliiflora* and highlight its potential as a promising source of bioactive molecules for the prevention and treatment of neuroinflammatory and neurodegenerative disorders. Furthermore, this work expands the known repertoire of bioactive compounds in *Magnolia* species by identifying spirocyclohexadienone-type lignans as key contributors to anti-inflammatory activity, a structural class not previously associated with this pharmacological property. Additional investigations, including in vivo efficacy studies and detailed mechanistic evaluations, will be required to validate these findings. Overall, the demonstrated activity of *M. liliiflora* compounds, both in crude extracts and as purified molecules, provides preliminary evidence supporting their further investigation as potential anti-neuroinflammatory materials.

## Figures and Tables

**Table 1:** ^1^H and ^13^C NMR data of new compounds **1** and **2** in CDCl_3_.

Position	1		2	
	*δ*_H_ (*J* in Hz)	*δ*_C_	*δ*_H_ (*J* in Hz)	*δ*_C_
1		136.3		134.2
2	6.38 s	106.3	6.65 s	103.1
3		153.2		147.1
4		136.8		136.2
5		153.2		147.1
6	6.38 s	106.3	6.65 s	103.1
7	2.01 t, (12.4) 3.38 dd, (12.3, 1.4)	37.7	4.45 dd, (6.6, 2.1)	87.4
8	2.31 m	43.4	2.30 m	44.4
9	0.60 d (6.8)	13.8	1.00 d (6.3)	12.9
10	3.85 s	56.3	3.90 s	56.4
11	3.82 s	61.0	3.90 s	56.4
12	3.85 s	56.3		
1′		141.7		133.4
2′	6.33 s	138.7	6.97 s	106.8
3′		80.5		147.9
4′		173.3		147.2
5′	5.74 s	105.6	6.78 d, (7.9)	147.2
6′		186.5	6.87 dd, (7.9, 1.7)	120.0
7′	3.16 m	33.2	4.45 dd, (6.6, 2.1)	87.4
8′	5.90 m	135.3	2.30 m	44.4
9′	5.16 dd, (5.6, 1.7) 5.13 t, (1.4)	117.3	1.03 d, (6.3)	12.9
10′	3.18 s	52.9	5.95 s	101.1
11′	3.78 s	56.2		
4-OH			5.46 s	

**Table 2:** Candidate bioactivity-associated markers in the EtOAc fraction of *M. liliiflora*.

*m*/*z*	RT (min)	Correlation (Spearman)	*p*-Value	FDR *
387.1833	8.27	0.87	0.00	0.01
343.1570	6.85	0.79	0.00	0.02
341.1415	9.20	0.79	0.00	0.02
266.1192	5.41	0.71	0.01	0.03
233.0825	9.05	0.70	0.01	0.04
278.0836	4.08	0.69	0.01	0.04

* Candidate markers were selected based on significant positive Spearman rank correlations between UPLC–MS/MS feature intensities and nitric oxide (NO) inhibition across *Magnolia liliiflora* EtOAc subfractions and reference *Magnolia* species (FDR < 0.05). Putative annotations were assigned based on MS/MS spectral similarity, molecular networking, and subsequent isolation and structure elucidation. RT, retention time.

**Table 3:** Selected pharmacokinetic properties predicted for isolated compounds.

Compound	GI Absorption	Blood–Brain Barrier Permeant	P-gp Substrate	Solubility
**1**	high	yes	no	moderately soluble
**2**	high	yes	no	moderately soluble
**3**	high	yes	no	moderately soluble
**4**	high	yes	no	moderately soluble
**5**	high	yes	no	moderately soluble
**6**	high	yes	no	moderately soluble
**7**	high	yes	yes	moderately soluble

## Data Availability

The data presented in this study are available on request from the corresponding author.

## Supplementary Materials

The following supporting information can be downloaded at: https://www.mdpi.com/article/10.3390/nu18111749/s1, Figure S1. HRESI-MS spectrum of compound **1**. Figure S2. IR spectrum of compound **1**. Figure S3. ^1^H NMR spectrum of compound **1** in chloroform-*d3*. Figure S4. ^13^C NMR spectrum of compound **1** in chloroform-*d*. Figure S5. COSY spectrum of compound **1** in chloroform-*d*. Figure S6. eHSQC spectrum of compound **1** in chloroform-d. Figure S7. HMBC spectrum of compound **1** in chloroform-*d*. Figure S8. NOESY spectrum of compound **1** in chloroform-*d*. Figure S9. 1D-NOESY spectrum of compound **1** in chloroform-*d*. Figure S10. UV spectrum of compound **1**. Figure S11. ECD spectrum of compound **1**. Figure S12. HRESI-MS spectrum of compound **2**. Figure S13. IR spectrum of compound **2**. Figure S14. ^1^H NMR spectrum of compound **2** in chloroform-*d*. Figure S15. ^13^C NMR spectrum of compound **2** in chloroform-*d*. Figure S16. COSY spectrum of compound **2** in chloroform-*d*. Figure S17. eHSQC spectrum of compound **2** in chloroform-*d*. Figure S18. HMBC spectrum of compound **2** in chloroform-*d*. Figure S19. NOESY spectrum of compound **2** in chloroform-*d*. Figure S20. UV spectrum of compound **2**. Figure S21. ECD spectrum of compound **2**. Figure S22. ^1^H and ^13^C NMR spectra of compound **3** in chloroform-*d*. Figure S23. ^1^H and ^13^C NMR spectra of compound **4** in chloroform-*d*. Figure S24. ^1^H and ^13^C NMR spectra of compound **5** in chloroform-*d*. Figure S25. ^1^H and ^13^C NMR spectra of compound **6** in chloroform-*d*. Figure S26. ^1^H and ^13^C NMR spectra of compound **7** in chloroform-*d*. Figure S27. NO inhibition and cell viability of several fractions of the 70% EtOH extract of *M. liliiflora* leaf. Figure S28. Clustering based on metabolomic profile of *Magnolia* spp. Figure S29. UHPLC-MS/MS of EtOAc fraction of 70% EtOH extract of *M. liliiflora* leaf. Figure S30. Molecular network cluster containing lignans. Figure S31. Molecular network cluster containing anonaine, biomarker candidate. Figure S32. Gene ontology enrichment of bioactive compounds predicted targets (from STRING database). Figure S33. Predicted target interaction network of the isolated active compounds. Figure S34. Molecular docking results of compound **1** in predicted targets IKKβ and TAB1-TAK1/2. Figure S35. Boiled-Egg diagram representing the predicted permeability of isolated compounds. Table S1. NO production inhibition by partition fractions of *M. liliiflora* Table S2. NO production inhibition by EtOAc subfractions of *M. liliiflora*. Table S3. NO production inhibition by EtOAc fractions of related *Magnolia* spp. Table S4. Summary of NO inhibition activity of Magnolia species and *Magnolia liliiflora* fractions. Table S5. Annotated features of *M. liliiflora* using GNPS library. Table S6. Biomarker candidate annotation. Table S7. Molecular docking results on Inflammatory-related targets (-CDOCKER values).
