# Supplementary material for: Functional Food Potential of Magnolia liliiflora Leaves: Chemical Profiling of Bioactive Lignans and Their Anti-Inflammatory Effects in LPS-Activated Microglia
Source: Nutrients. 2026 May 29;18(11):1749. doi: 10.3390/nu18111749 (PMC13258255; doi:10.3390/nu18111749)
Supplement: Supplementary file 1 [file nutrients-18-01749-s001.zip › nutrients-4302606-supplementary.pdf]

Supporting Information

Functional Food Potential of *Magnolia liliiflora* Leaves: Chemical Profiling of Bioactive Lignans and Their Anti-Inflammatory Effects in LPS-Activated Microglia

Jorge-Eduardo Ponce-Zea [1], Yun-Hui Che [1], Gwan-Young Jung [1], Van-Hieu Mai [1], Minh-Thi-Tuyet Le [1], Jin-Pyo An [2] and Won-Keun Oh [1,*]

[1] Research Institute of Pharmaceutical Sciences, College of Pharmacy, Seoul National University, Seoul 08826, Republic of Korea; jepz210689@snu.ac.kr (J.-E.P.-Z.); woonhye419@snu.ac.kr (Y.-H.C.); rhksdud9951@snu.ac.kr (G.-Y.J.); maihieu@snu.ac.kr (V.-H.M.); lethituyetminh19289@gmail.com (M.-T.-T.L.)

[2] Department of Industrial Crop Science and Technology, College of Agriculture, Life & Environment Sciences, Chungbuk National University, Cheongju 28644, Republic of Korea; anjinpyo@chungbuk.ac.kr

[*] Correspondence: wkoh1@snu.ac.kr; Tel.: +82-2-880-7872

List of Supporting information

Figure S1. HRESI(+) MS spectrum of compound 1 4

Figure S2. IR spectrum of compound 1 4

Figure S3. ^{1}H NMR spectrum of compound 1 in chloroform-*d* 5

Figure S4. ^{13}C NMR spectrum of compound 1 in chloroform-*d* 5

Figure S5. COSY spectrum of compound 1 in chloroform-*d* 6

Figure S6. eHSQC spectrum of compound 1 in chloroform-*d* 6

Figure S7. HMBC spectrum of compound 1 in chloroform-*d* 7

Figure S8. NOESY spectrum of compound 1 in chloroform-*d* 7

Figure S9. 1D-NOESY spectrum of compound 1 in chloroform-*d*. Irradiation at 2.32 ppm. ... 8

Figure S10. UV spectrum of compound 1 9

Figure S11. ECD spectrum of compound 1 9

Figure S12. HRESI(+)MS spectrum of compound 2 10

Figure S13. IR spectrum of compound 2 10

Figure S14. ^{1}H NMR spectrum of compound 2 in chloroform-*d* 11

Figure S15. ^{13}C NMR spectrum of compound 2 in chloroform-*d* 11

Figure S16. COSY spectrum of compound 2 in chloroform-*d* 12

Figure S17. eHSQC spectrum of compound 2 in chloroform-*d* 12

Figure S18. HMBC spectrum of compound 2 in chloroform-*d* 13

Figure S19. NOESY spectrum of compound 2 in chloroform-*d* 13

Figure S20. UV spectrum of compound 2 14

Figure S21. ECD spectrum of compound 2 14

Figure S22. ^{1}H, ^{13}C NMR spectra of compound 3 in chloroform-*d* 15

Figure S23. ^{1}H, ^{13}C NMR spectra of compound 4 in chloroform-*d* 16

Figure S24. ^{1}H, ^{13}C NMR spectra of compound 5 in chloroform-*d* 17

Figure S25. ^{1}H, ^{13}C NMR spectra of compound 6 in chloroform-*d* 18

Figure S26. ^{1}H, ^{13}C NMR spectra of compound 7 in chloroform-*d* 19

Figure S27. NO inhibition and cell viability of M. liliiflora leaf ethanolic extract fractions. .21

Figure S28. Clustering based on metabolomic profile of Magnolia spp.**Error! Bookmark not defined.**

Figure S29. UHPLC-MS/MS of EtOAc fraction of 70% EtOH extract of *M. liliiflora* leaf .... **Error! Bookmark not defined.**

Figure S30. Molecular network cluster containing lignans 22

Figure S31. Molecular network cluster containing anonaine, biomarker candidate........ **Error! Bookmark not defined.**

Figure S32. Gene ontology enrichment of bioactive compounds predicted targets (from STRING database). 24

Figure S33. Predicted target interaction network of the isolated active compounds...............25

Figure S34. Molecular docking results of compound 1 in predicted targets IKKβ and TAB1-TAK1/2. 26

Figure S35. Boiled-Egg diagram representing the predicted permeability of isolated compounds. 27

Table S1. Effects of *Magnolia liliiflora* leaf fractions on nitric oxide production in LPS-stimulated BV2 microglial cells...28

Table S2. Inhibitory effects of EtOAc subfractions of *Magnolia liliiflora* leaves on nitric oxide production in LPS-stimulated BV2 microglial cells 29

Table S3. Comparison of nitric oxide inhibitory effects of EtOAc fractions from *Magnolia* species in LPS-stimulated BV2 microglial cells...30

Table S4 Summary of NO inhibition activity of Magnolia species and *Magnolia liliiflora* fractions...31

Table S5. Annotated features of *M. liliiflora* using GNPS library 32

Table S6. Biomarkers candidate annotation 39

Table S7. Molecular docking results on Inflammatory related targets (-CDOCKER values).40

Figure S1. HRESI(+) MS spectrum of compound **1**

Elemental Composition Report Page 1

Tolerance = 20.0 PPM / DBE: min = -1.5, max = 50.0
Element prediction: Off
Number of isotope peaks used for i-FIT = 3

Monoisotopic Mass, Even Electron Ions
776 formula(e) evaluated with 12 results within limits (all results (up to 1000) for each mass)
Elements Used:
C: 0-500 H: 0-1000 N: 0-10 O: 0-20

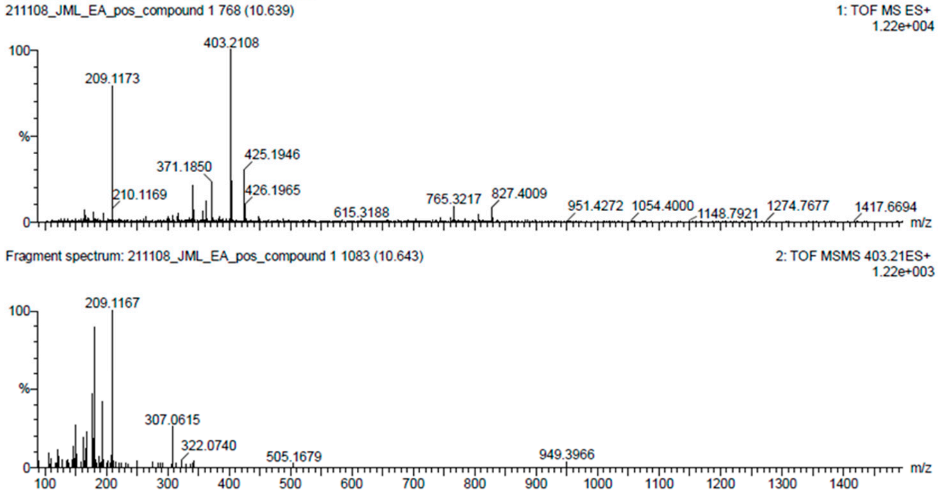

Minimum:	80.00				-1.5				
Maximum:	100.00		5.0	20.0	50.0				
Mass	RA	Calc. Mass	mDa	PPM	DBE	i-FIT	Norm	Conf(%)	Formula
403.2108	100.00	403.2094	1.4	3.5	9.5	95.9	0.055	94.63	C19 H27 N6 O4
		403.2107	0.1	0.2	14.5	99.2	3.366	3.45	C20 H23 N10
		403.2121	-1.3	-3.2	8.5	100.6	4.831	0.80	C23 H31 O6
		403.2080	2.8	6.9	4.5	100.7	4.902	0.74	C18 H31 N2 O8
		403.2134	-2.6	-6.4	13.5	102.2	6.381	0.17	C24 H27 N4 O2

Figure S2. IR spectrum of compound **1**

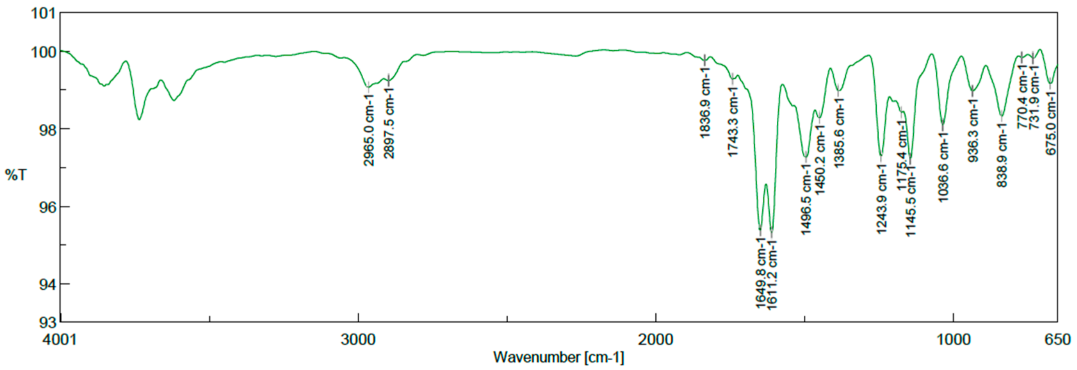

[Comment]
Sample Name MALI001
Comment
User PONCE
Division
Company Pharmsnu

[Data Information]
Creation Date 2025-05-02 오후 4:37
Data array type Linear data array
Horizontal Wavenumber [cm-1]
Vertical %T
Start 649.893 cm-1
End 4000.6 cm-1
Data pitch 0.964233 cm-1
Data points 3476

[Measurement Information]
Model Name FT/IR-4200typeA
Serial Number B038361018

Light Source Standard
Detector TGS
Accumulation 24
Resolution 4 cm-1
Zero Filling On
Apodization Cosine
Gain Auto (2)
Aperture Auto (7.1 mm)
Scanning Speed Auto (2 mm/sec)
Filter Auto (30000 Hz)

Figure S3. ^{1}H NMR spectrum of compound **1** in chloroform-***d***

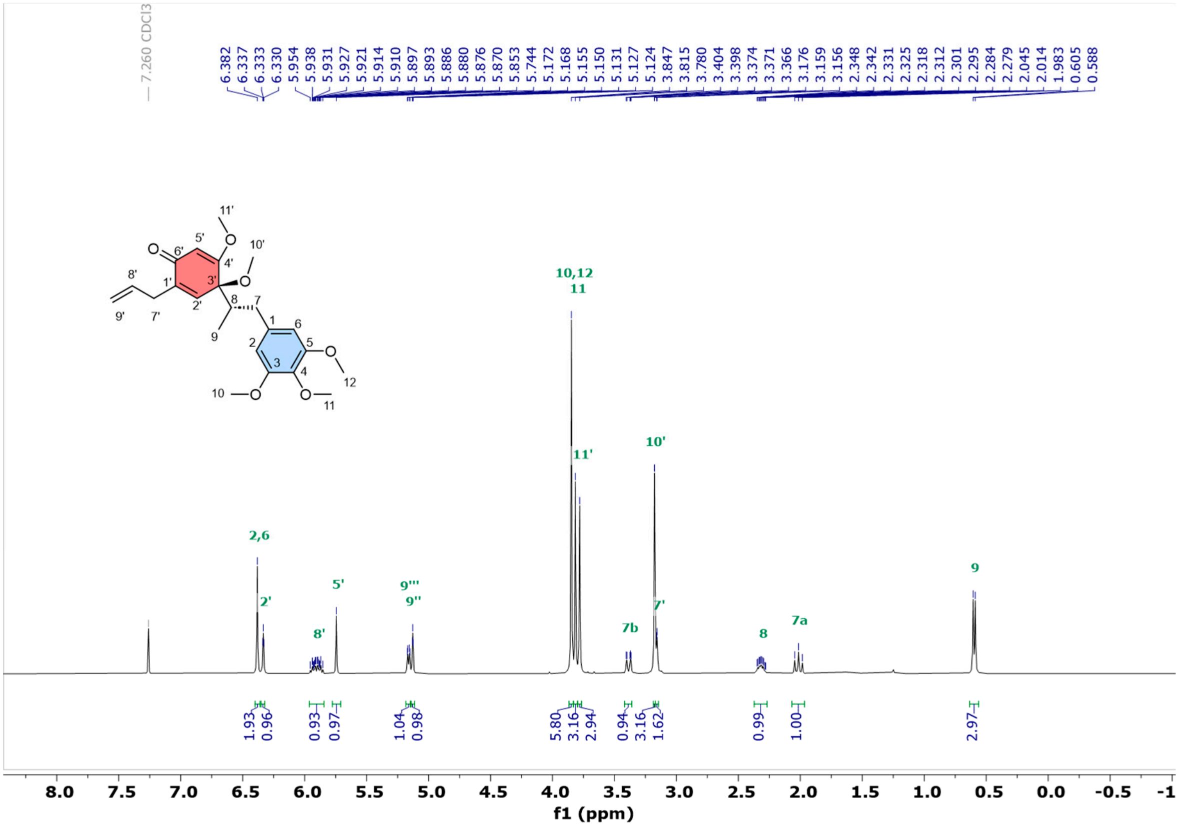

Figure S4. ^{13}C NMR spectrum of compound **1** in chloroform-*d*

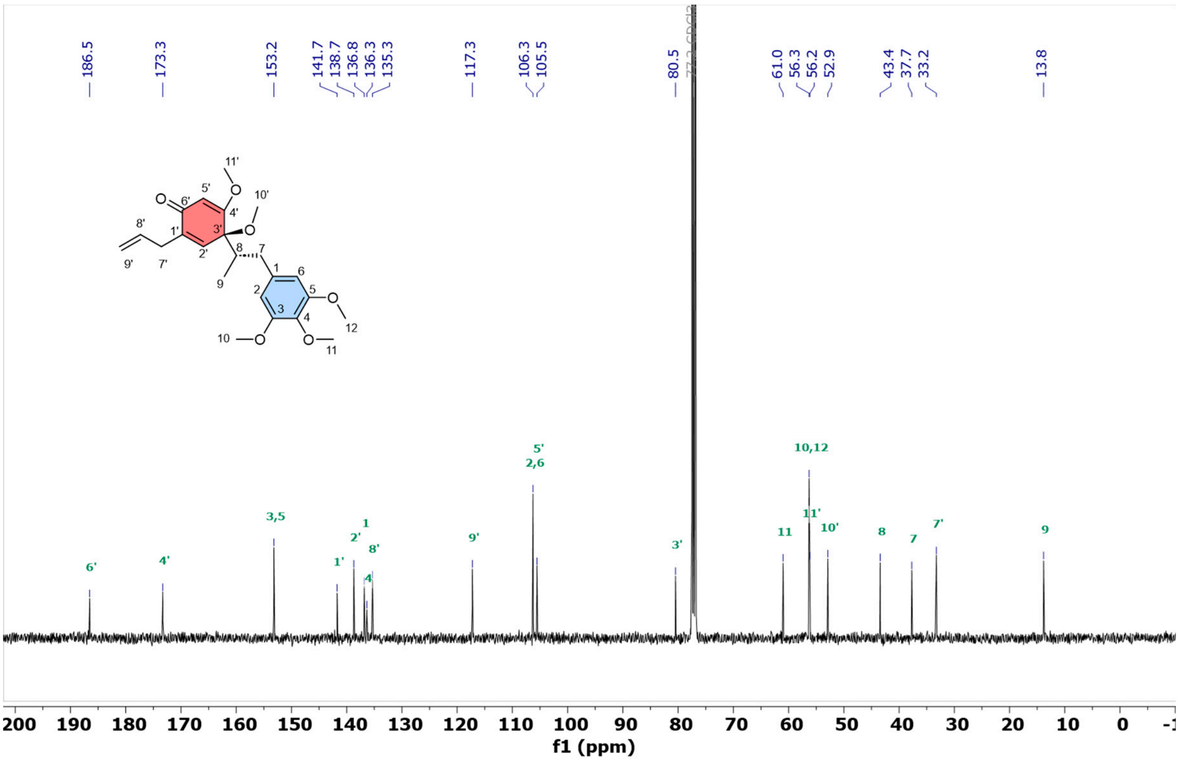

Figure S5. COSY spectrum of compound **1** in chloroform-*d*

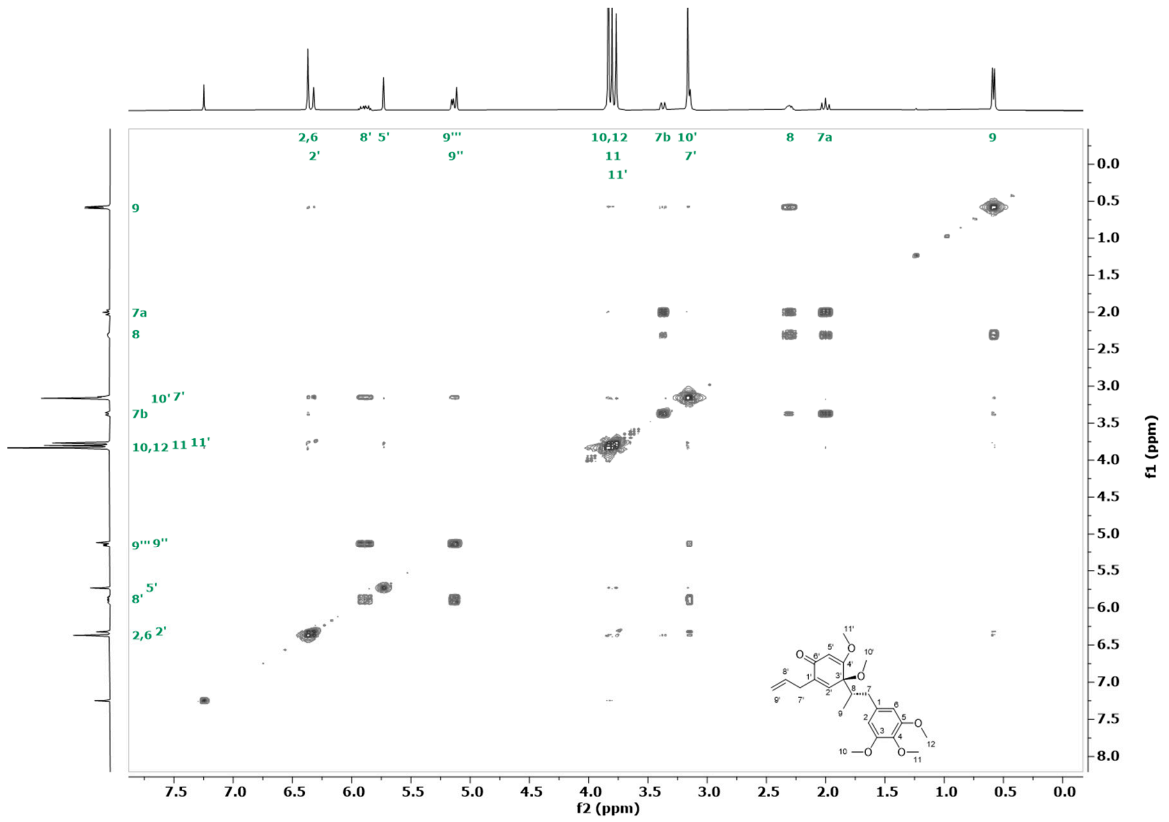

Figure S6. eHSQC spectrum of compound **1** in chloroform-*d*

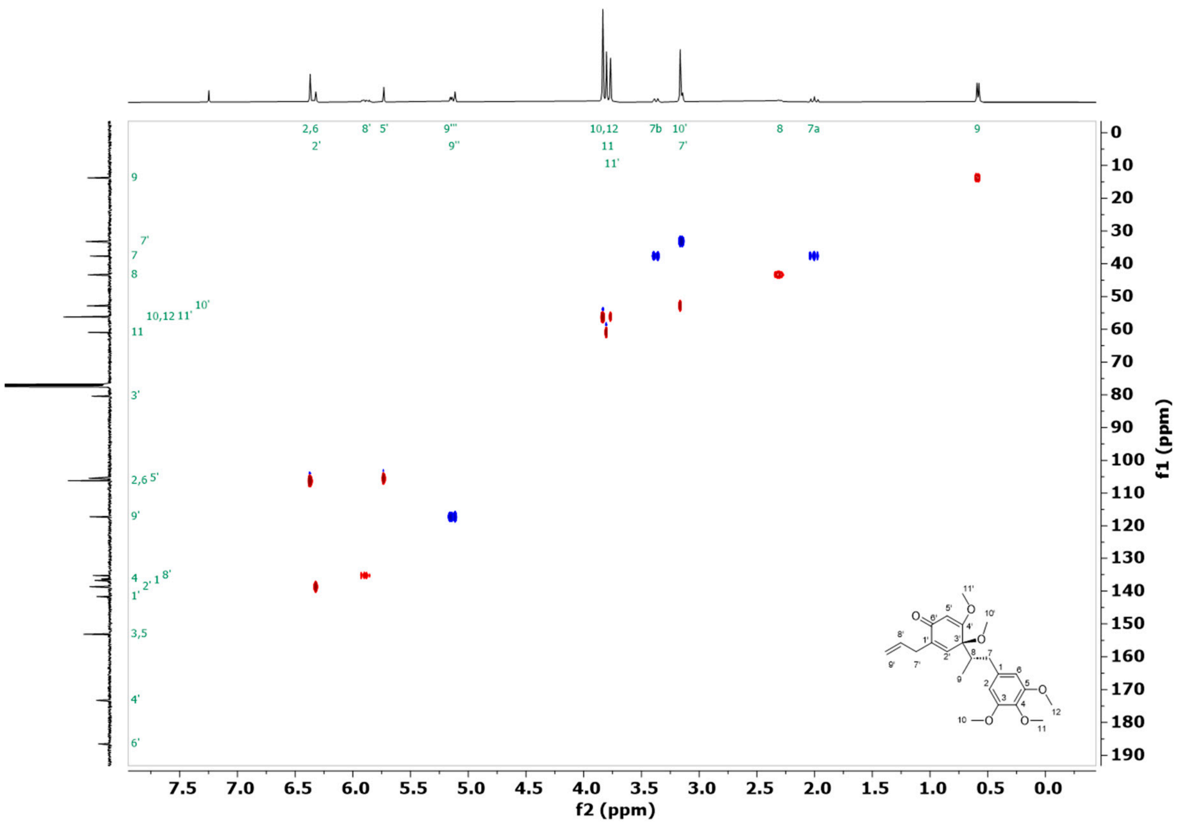

Figure S7. HMBC spectrum of compound **1** in chloroform-*d*

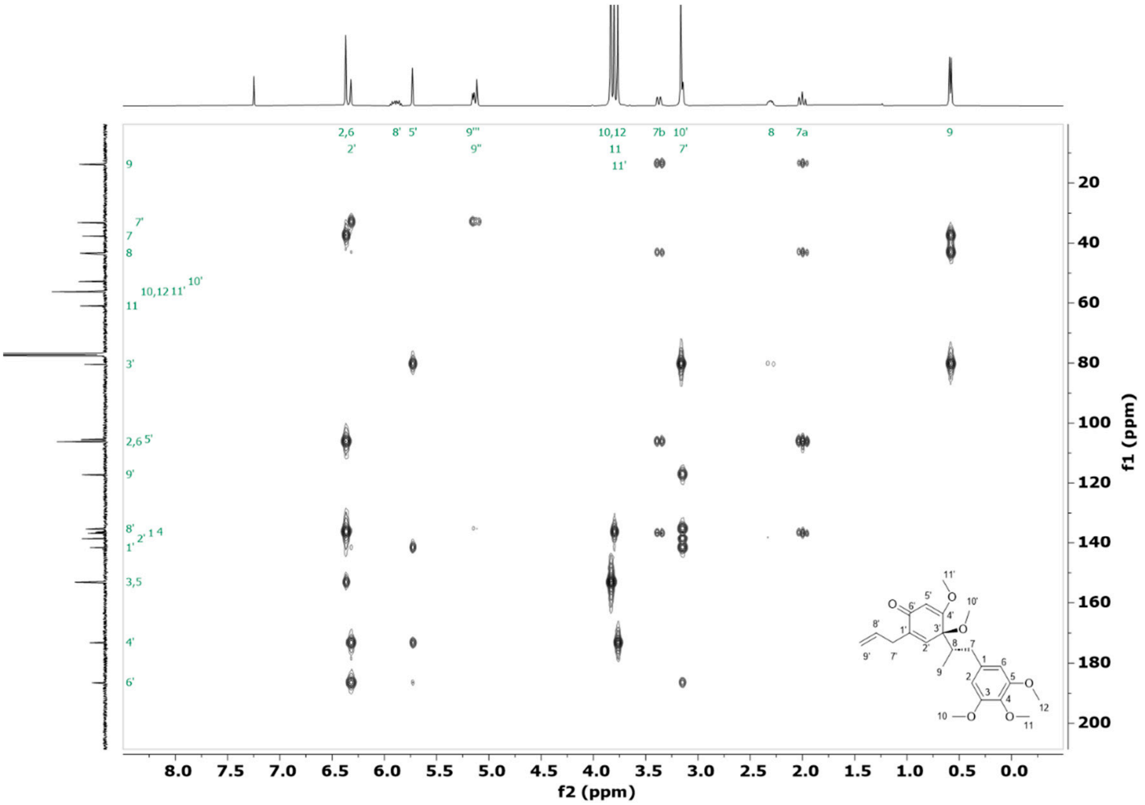

Figure S8. NOESY spectrum of compound **1** in chloroform-*d*

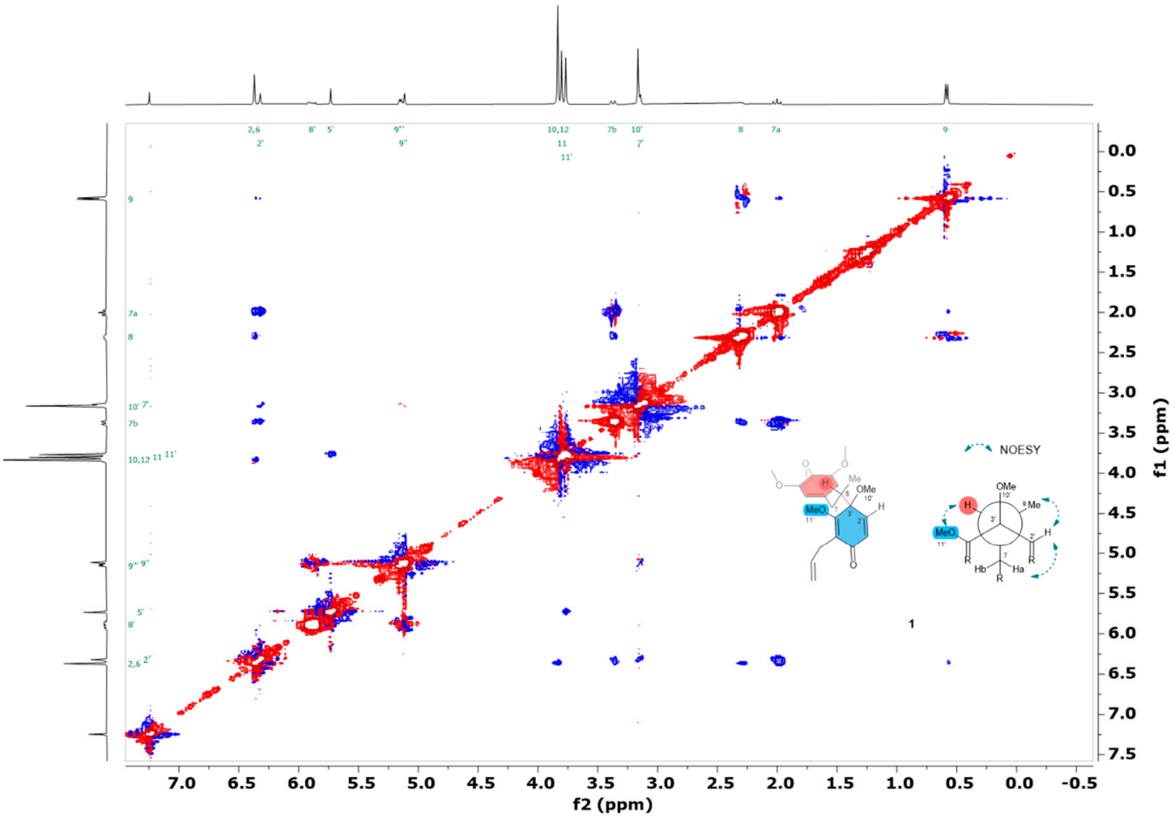

Figure S9. 1D-NOESY spectrum of compound **1** in chloroform-*d*. Irradiation at 2.32 ppm.

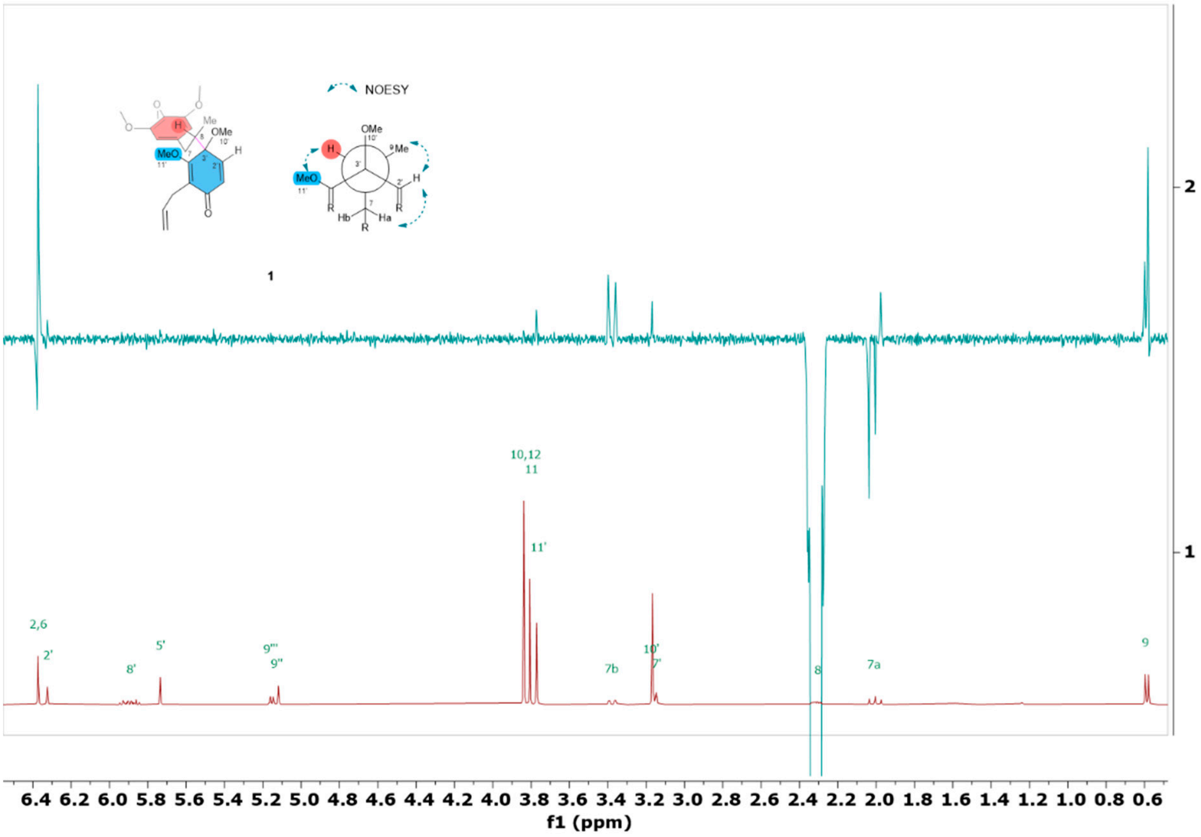

Figure S10. UV spectrum of compound **1**

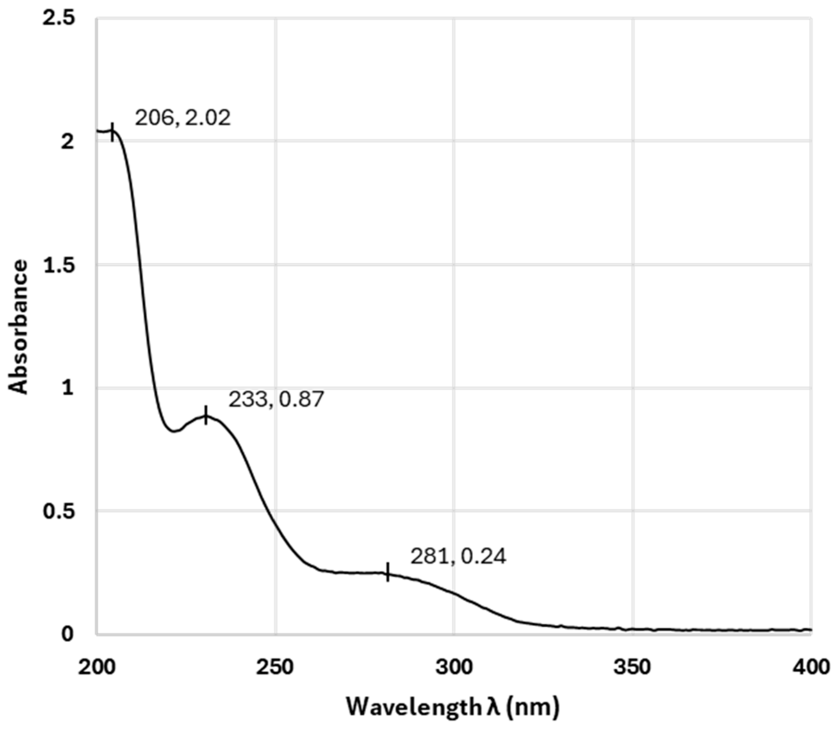

Figure S11. ECD spectrum of compound **1**

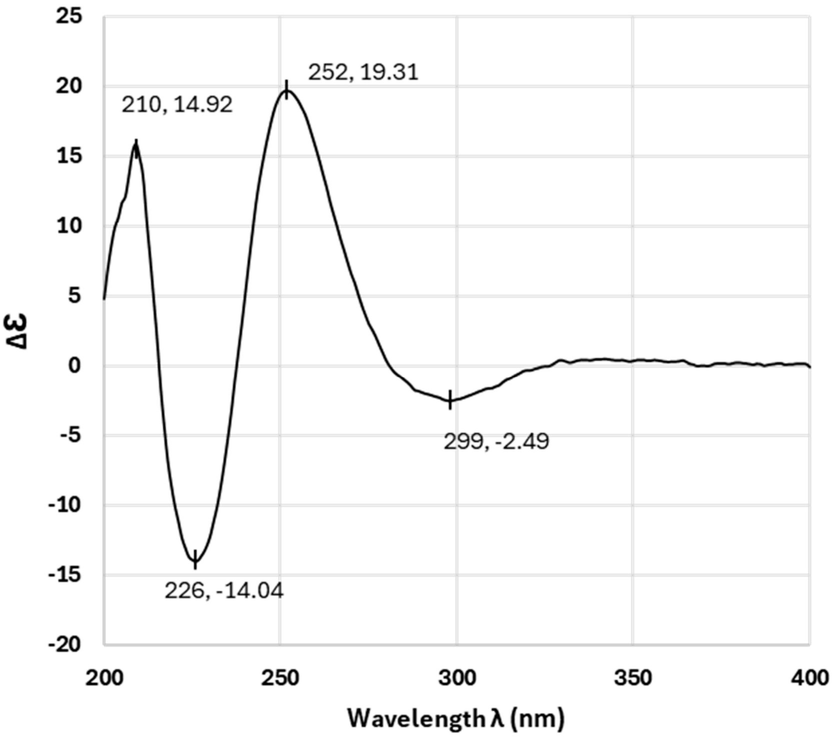

Figure S12. HRESI(+)MS spectrum of compound **2**

Elemental Composition Report

Page 1

Tolerance = 20.0 PPM / DBE: min = -1.5, max = 50.0
Element prediction: Off
Number of isotope peaks used for i-FIT = 3

Monoisotopic Mass, Even Electron Ions
416 formula(e) evaluated with 7 results within limits (all results (up to 1000)) for each mass)
Elements Used:
C: 0-500 H: 0-1000 N: 0-5 O: 0-20

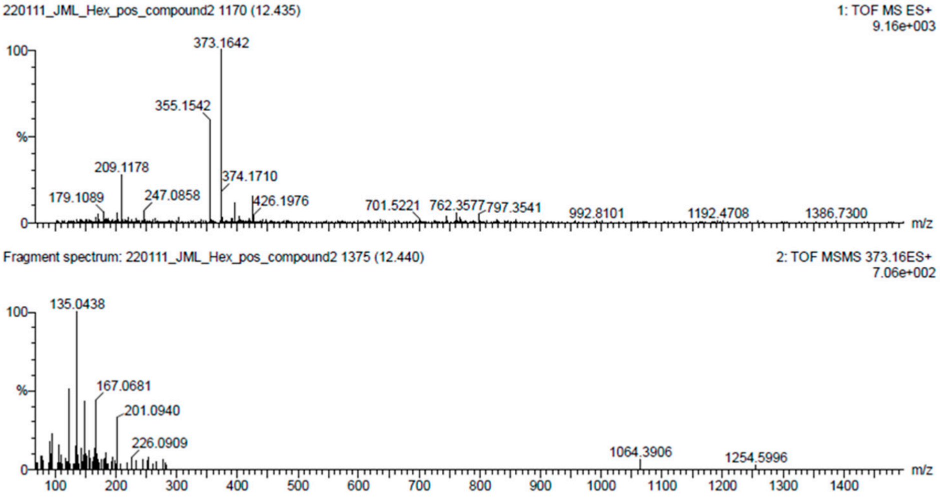

Minimum:	80.00				-1.5				
Maximum:	100.00		5.0	20.0	50.0				

Mass	RA	Calc. Mass	mDa	PPM	DBE	i-FIT	Norm	Conf(%)	Formula
373.1642	100.00	373.1651	-0.9	-2.4	9.5	107.3	5.661	0.35	C21 H25 O6
		373.1665	-2.3	-6.2	14.5	108.8	7.096	0.08	C22 H21 N4 O2
		373.1611	3.1	8.3	5.5	101.7	0.060	94.16	C16 H25 N2 O8
		373.1592	5.0	13.4	18.5	110.5	8.833	0.01	C28 H21 O
		373.1705	-6.3	-16.9	18.5	110.4	8.734	0.02	C27 H21 N2
		373.1710	-6.8	-18.2	0.5	104.7	3.000	4.98	C14 H29 O11
		373.1571	7.1	19.0	1.5	107.2	5.517	0.40	C11 H25 N4 O10

Figure S13. IR spectrum of compound **2**

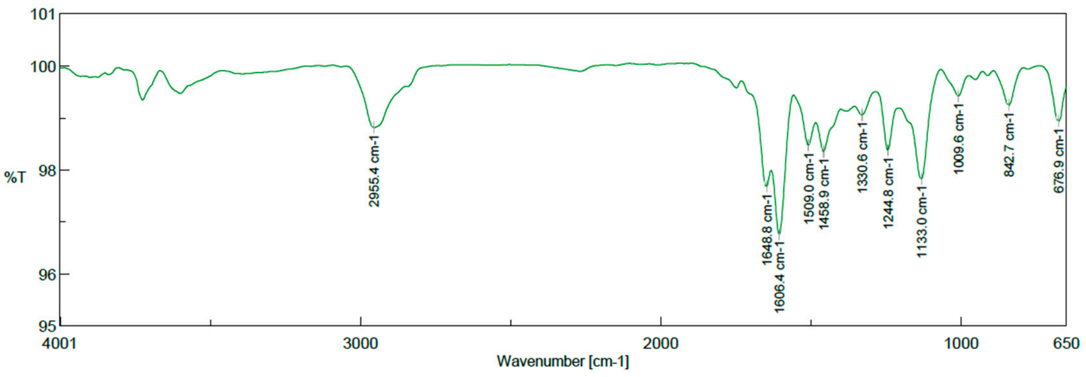

[Comment]
Sample Name MALI002
Comment
User PONCE
Division
Company Pharmsnu

[Data Information]
Creation Date 2025-05-02 오후 4:45
Data array type Linear data array
Horizontal Wavenumber [cm-1]
Vertical %T
Start 649.893 cm-1
End 4000.6 cm-1
Data pitch 0.964233 cm-1
Data points 3476

[Measurement Information]
Model Name FT/IR-4200typeA
Serial Number B038361018

Light Source Standard
Detector TGS
Accumulation 24
Resolution 4 cm-1
Zero Filling On
Apodization Cosine
Gain Auto (2)
Aperture Auto (7.1 mm)
Scanning Speed Auto (2 mm/sec)
Filter Auto (30000 Hz)

Figure S14. ^{1}H NMR spectrum of compound **2** in chloroform-*d*

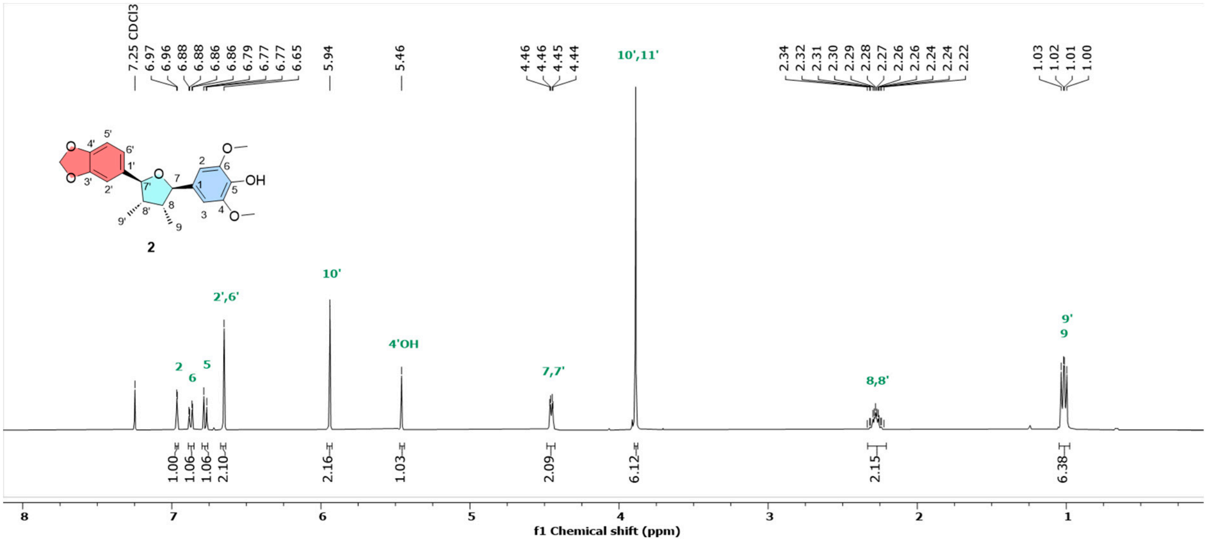

Figure S15. ^{13}C NMR spectrum of compound **2** in chloroform-*d*

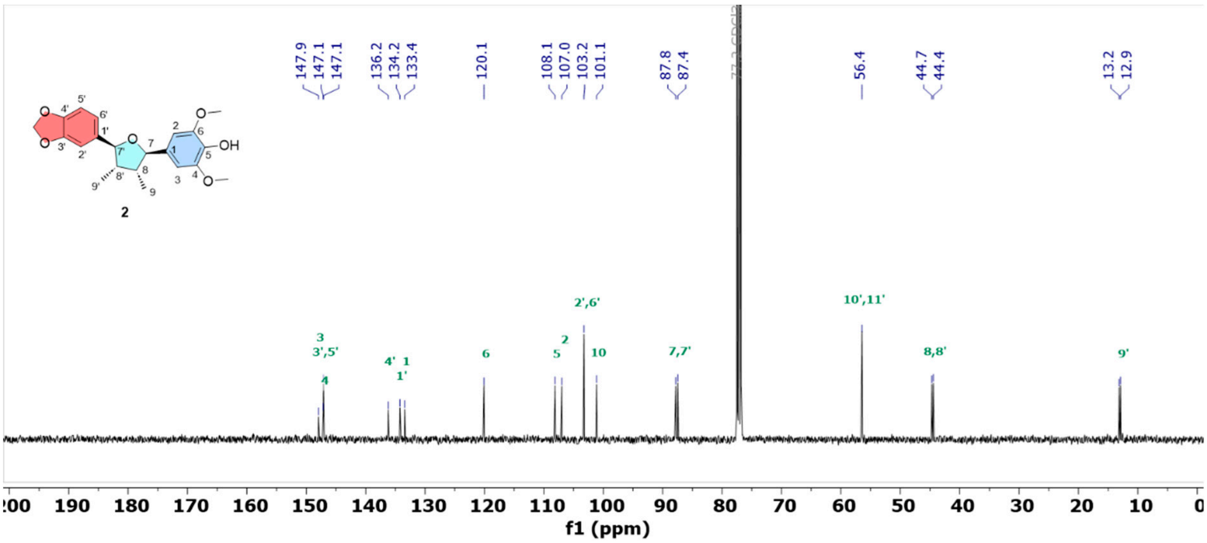

Figure S16. COSY spectrum of compound **2** in chloroform-*d*

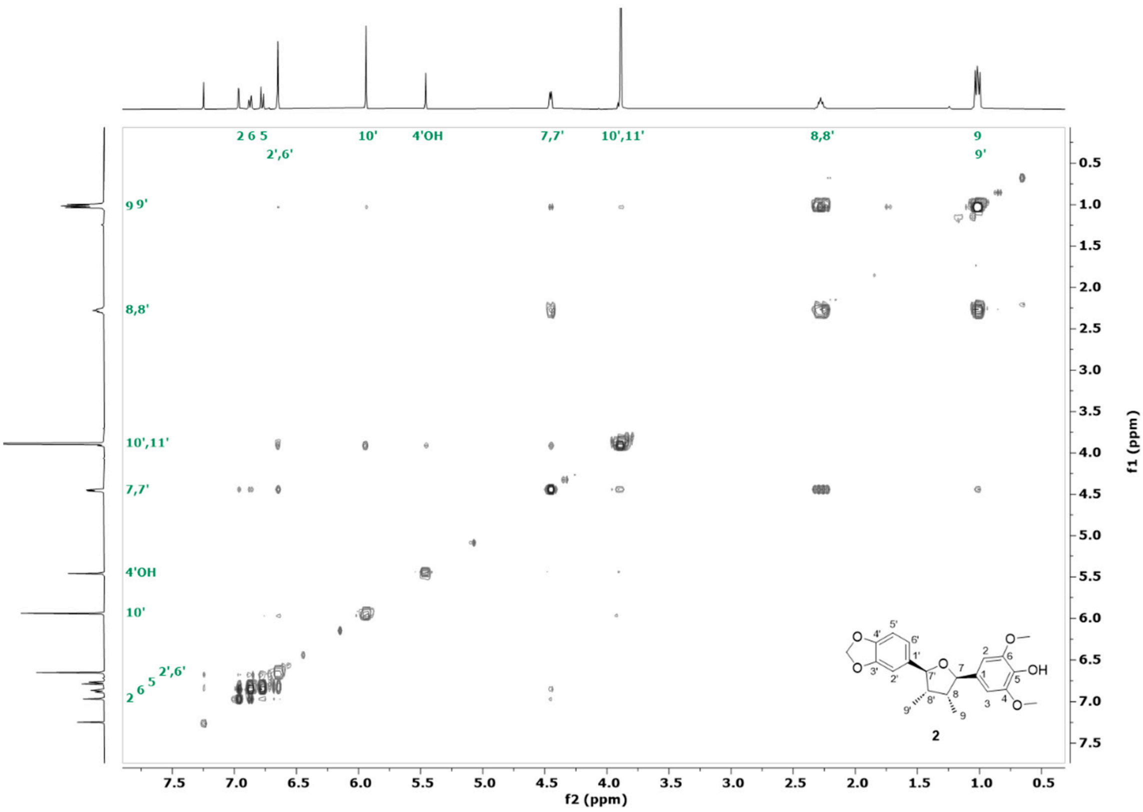

Figure S17. eHSQC spectrum of compound **2** in chloroform-*d*

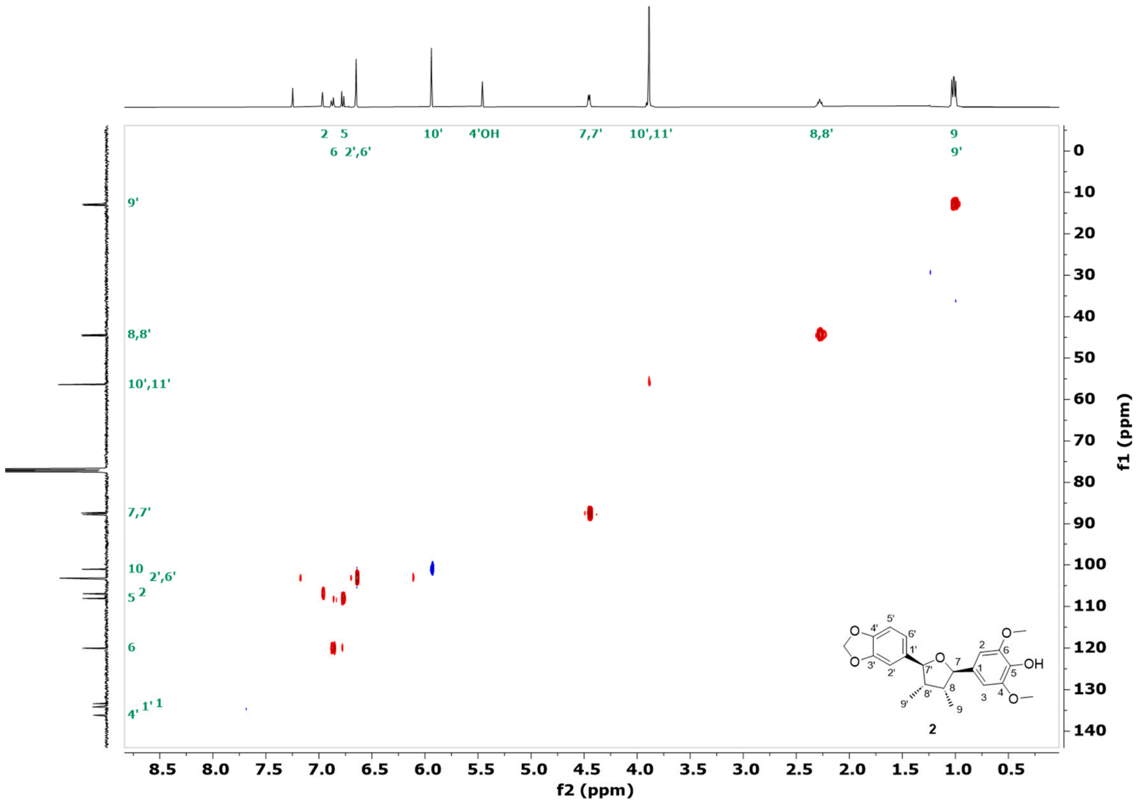

Figure S18. HMBC spectrum of compound **2** in chloroform-*d*

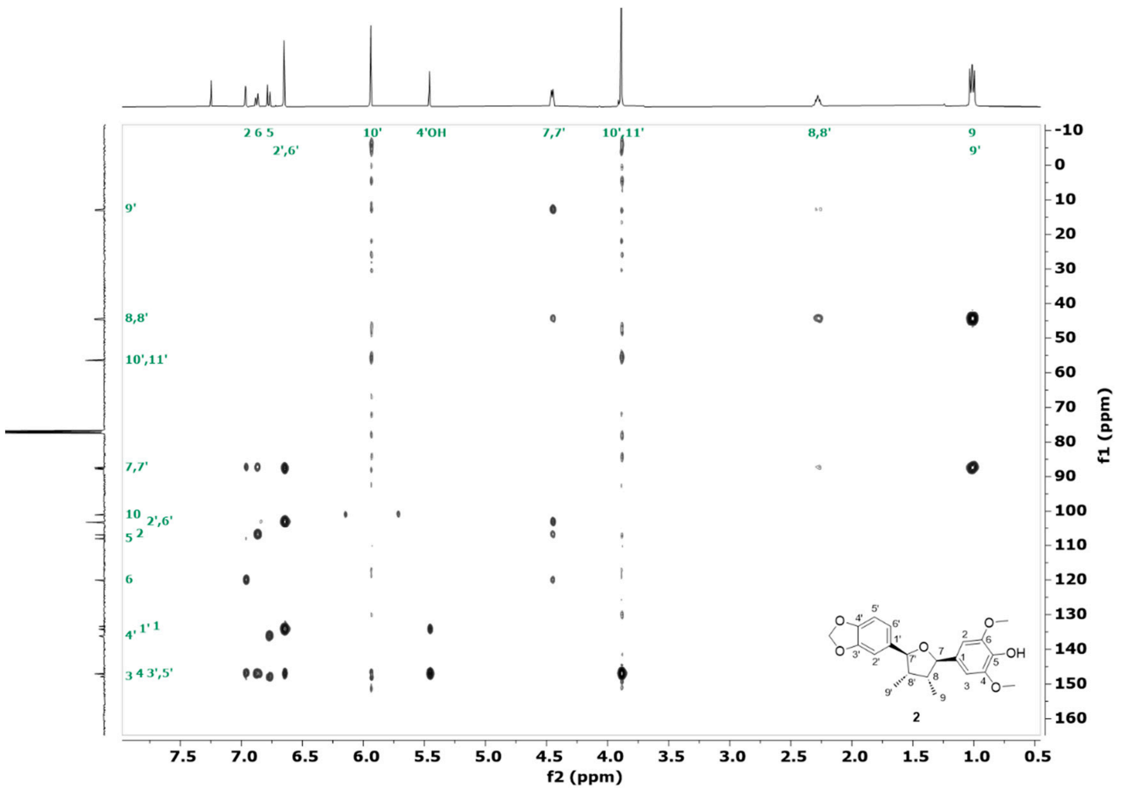

Figure S19. NOESY spectrum of compound **2** in chloroform-*d*

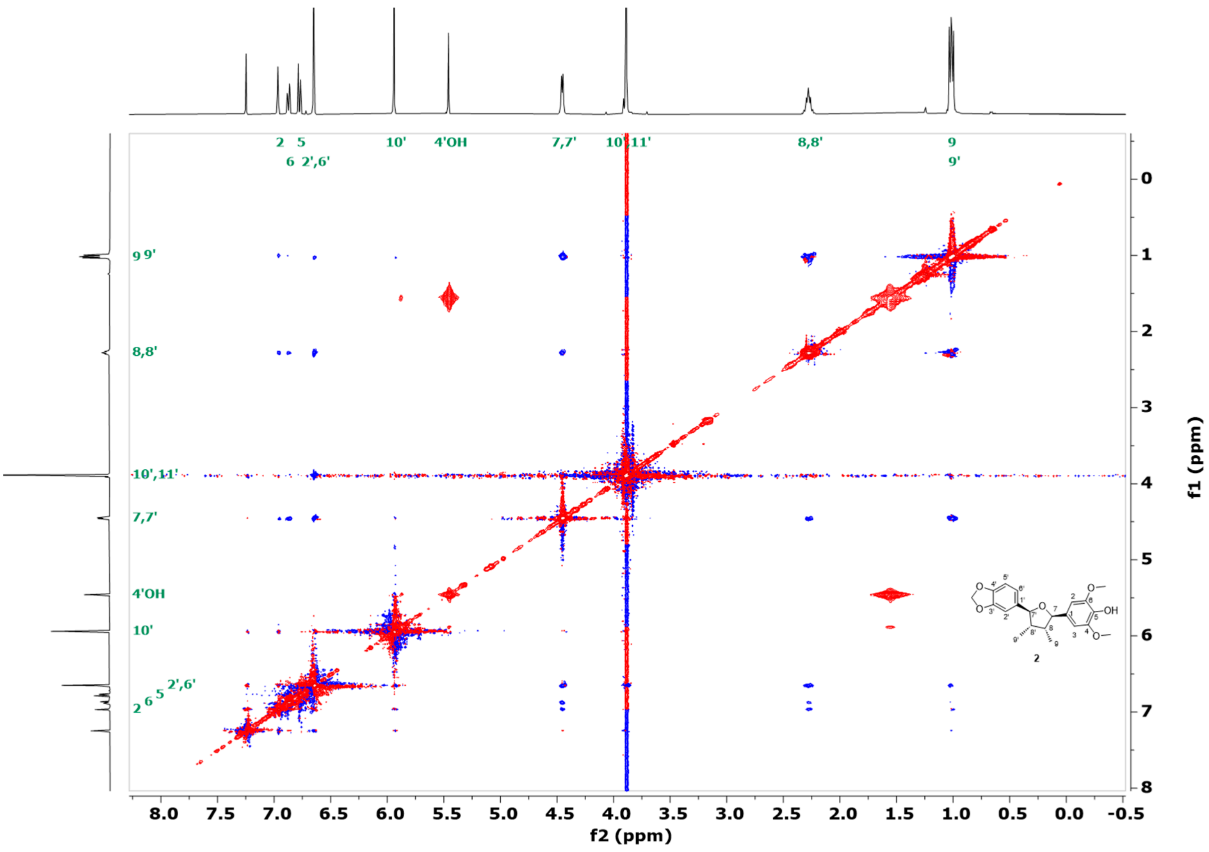

Figure S20. UV spectrum of compound **2**

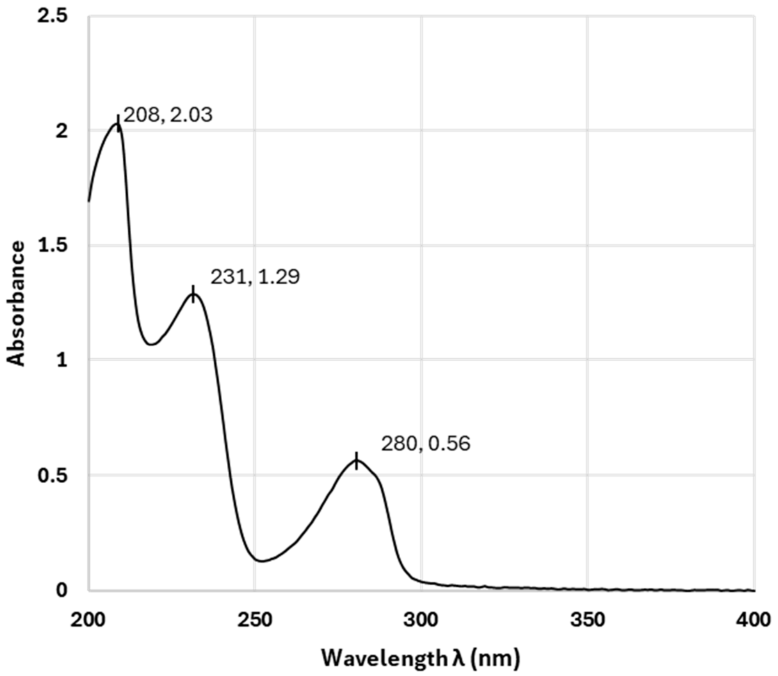

Figure S21. ECD spectrum of compound **2**

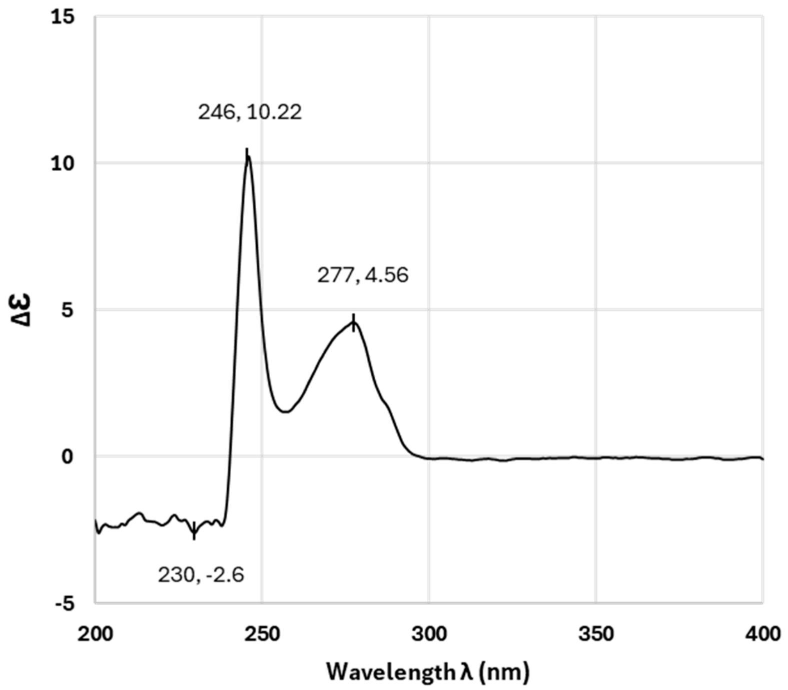

Figure S22. ^{1}H, ^{13}C NMR spectra of compound **3** in chloroform-*d*

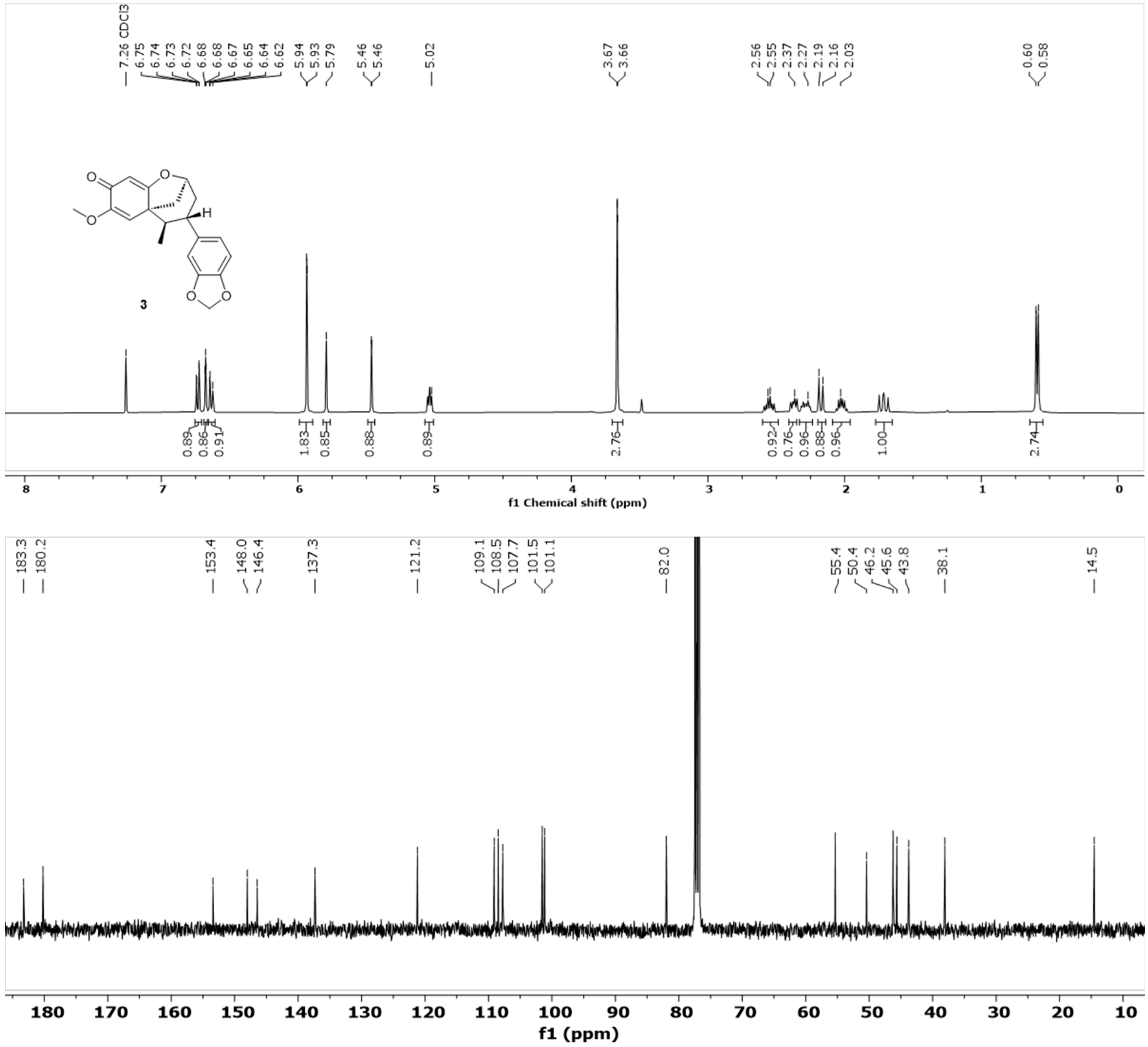

Figure S23. ^{1}H, ^{13}C NMR spectra of compound **4** in chloroform-*d*

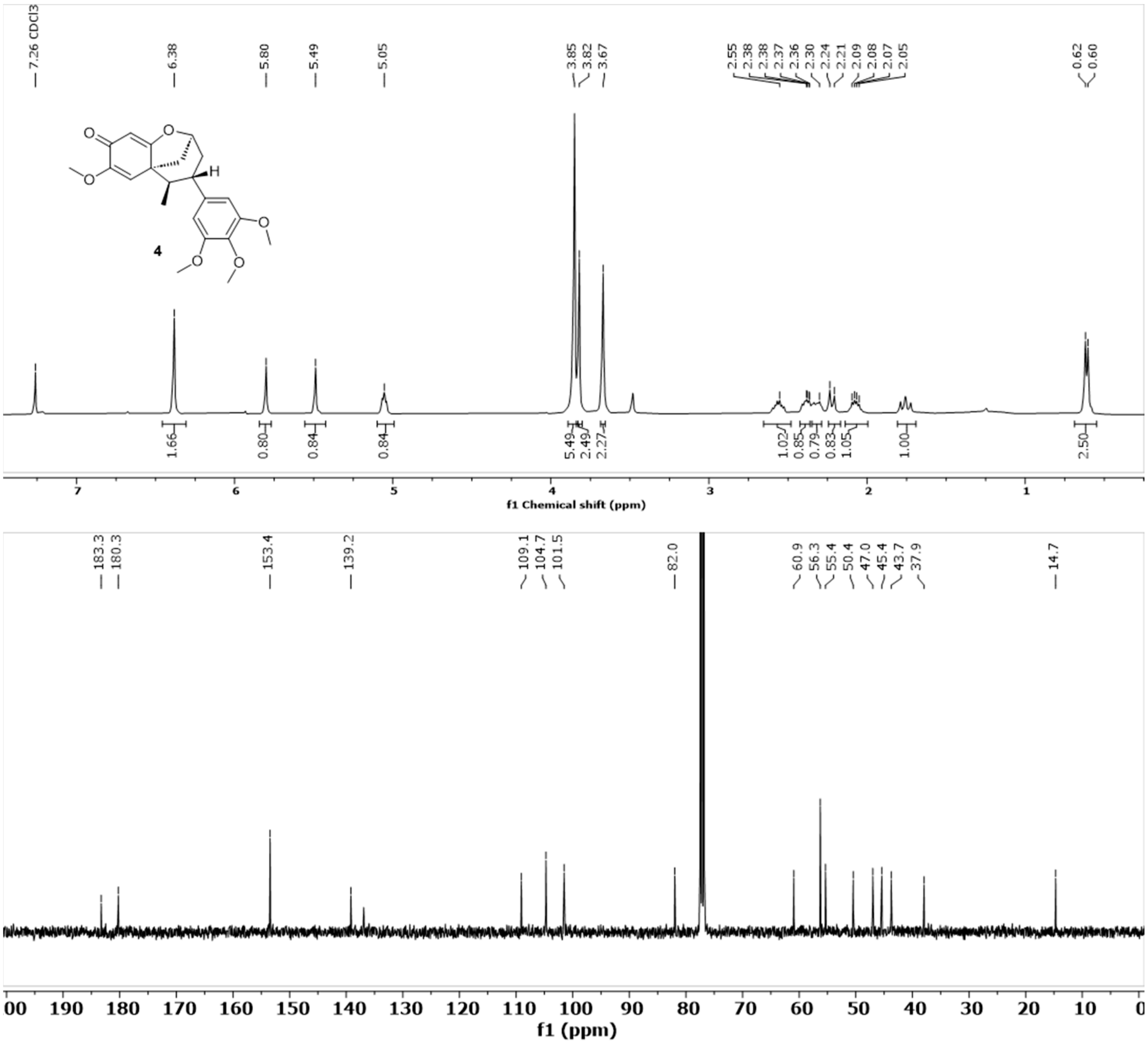

Figure S24. ^{1}H, ^{13}C NMR spectra of compound **5** in chloroform-*d*

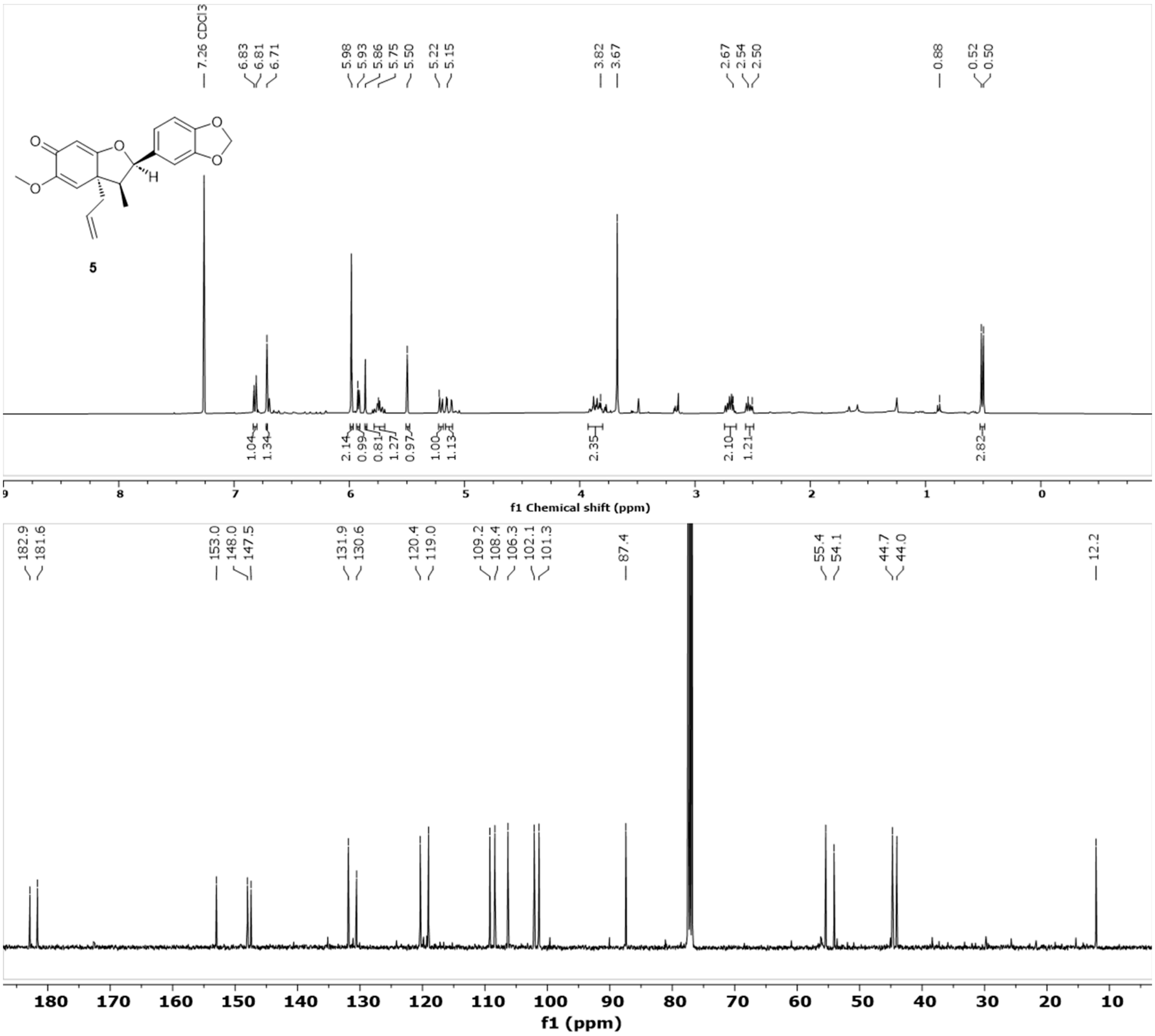

Figure S25. ^{1}H, ^{13}C NMR spectra of compound **6** in chloroform-*d*

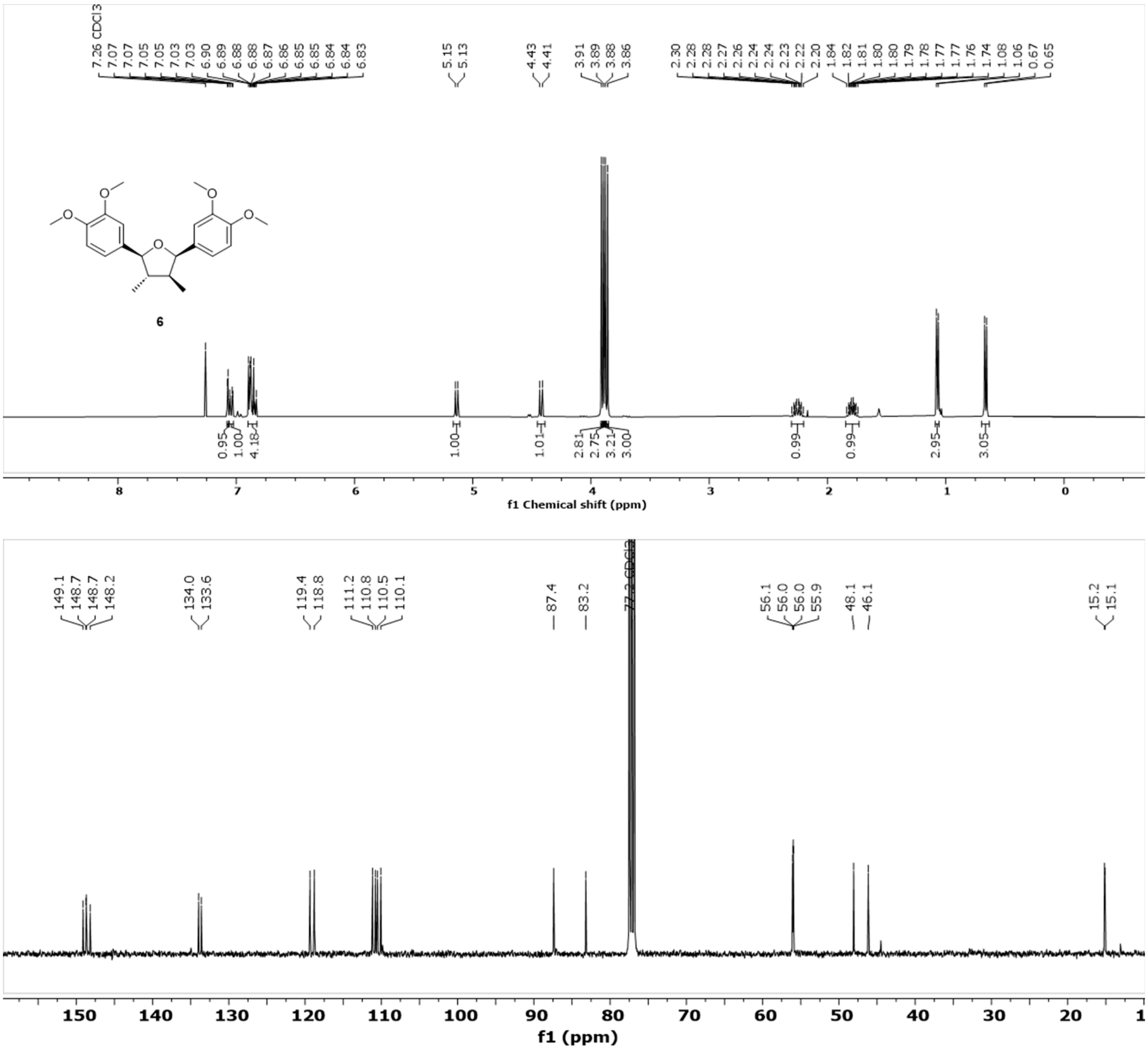

Figure S26. ^{1}H, ^{13}C NMR spectra of compound **7** in chloroform-*d*

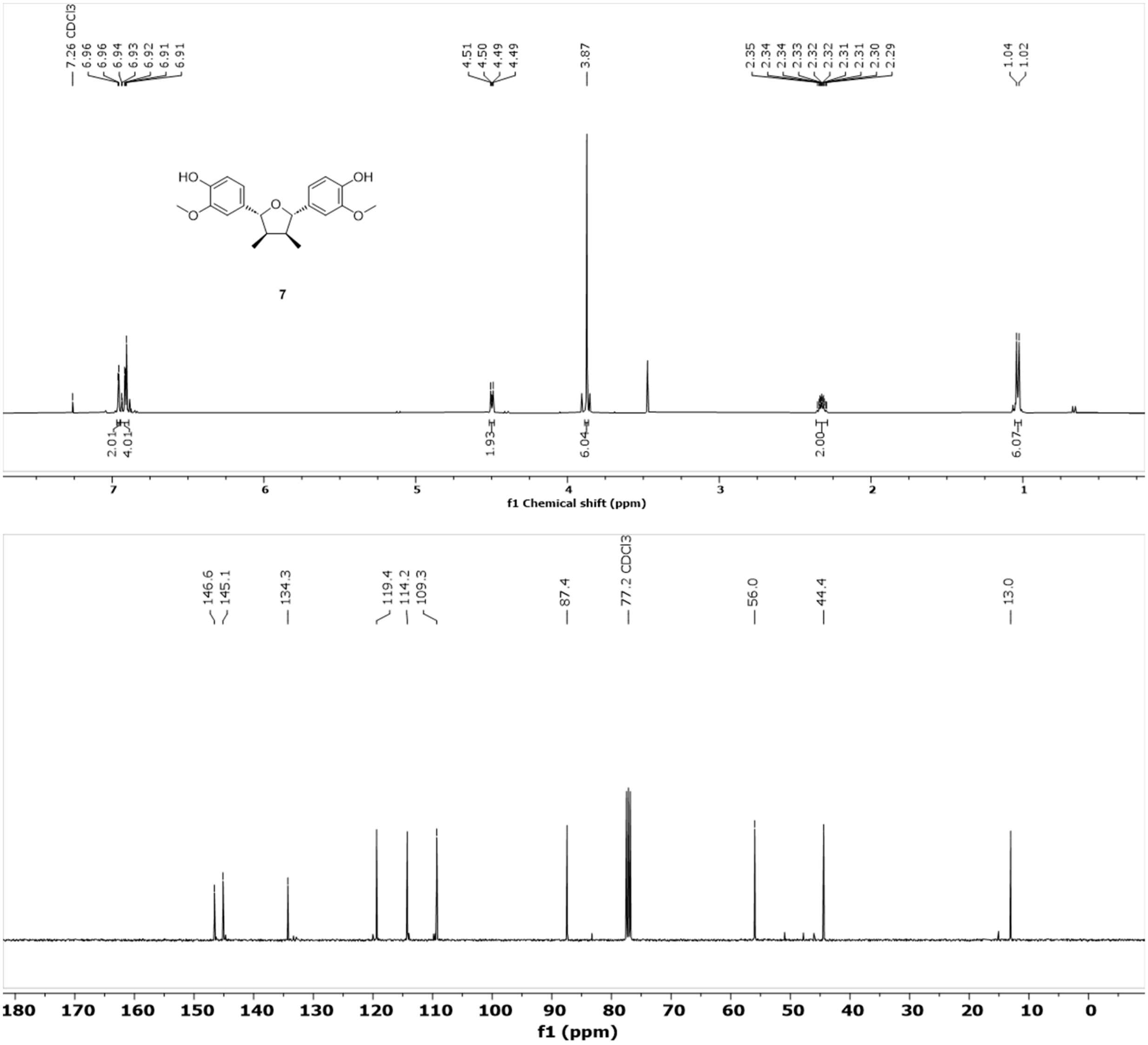

Figure S27. NO inhibition and cell viability of *M. liliiflora* leaf ethanolic extract fractions

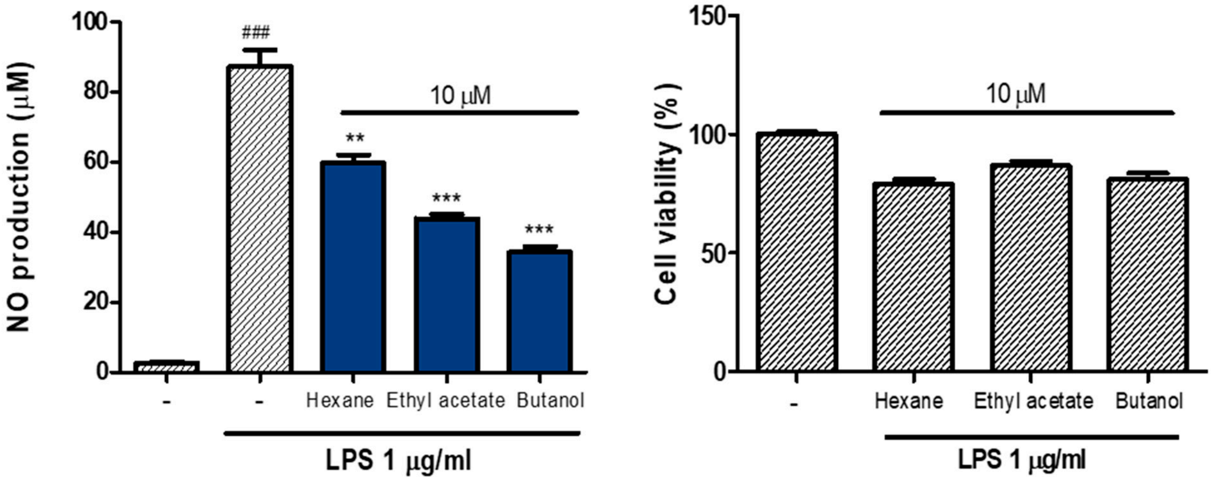

Figure S28. Clustering based on metabolomic profile of *Magnolia* spp.

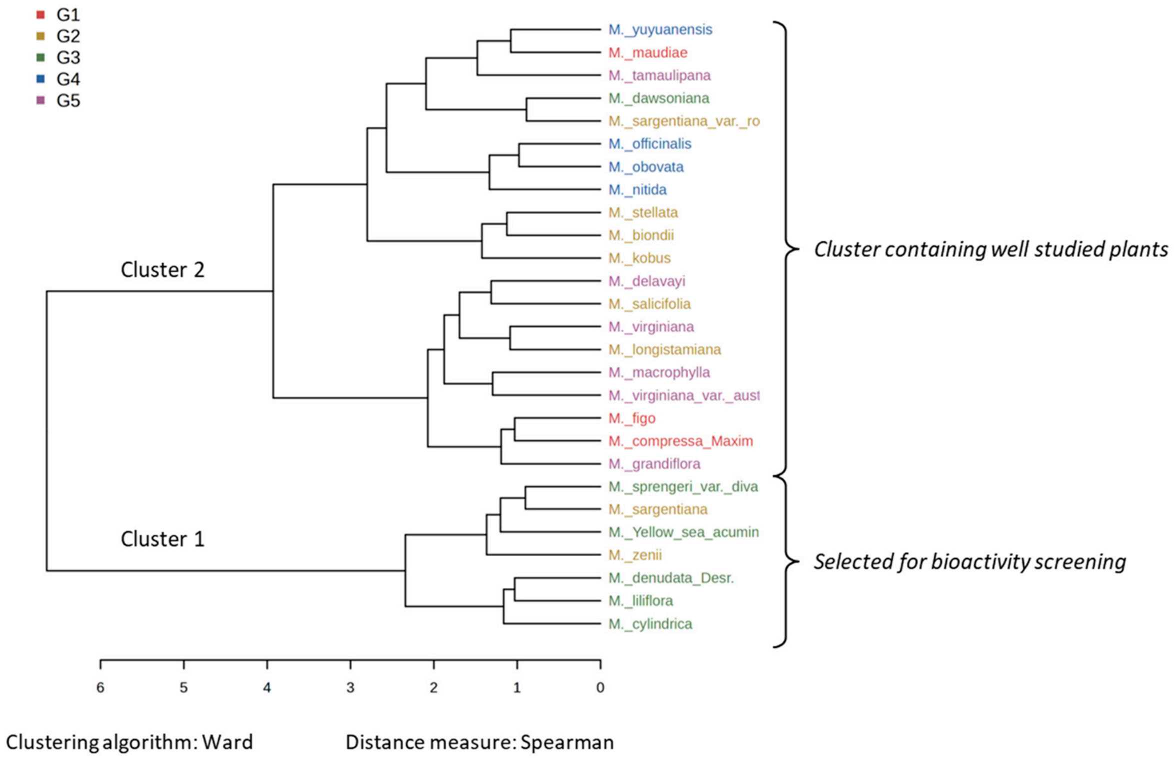

Figure S29. LC-MS/MS of EtOAc fraction of *M. liliiflora* leaf EtOH Extract.

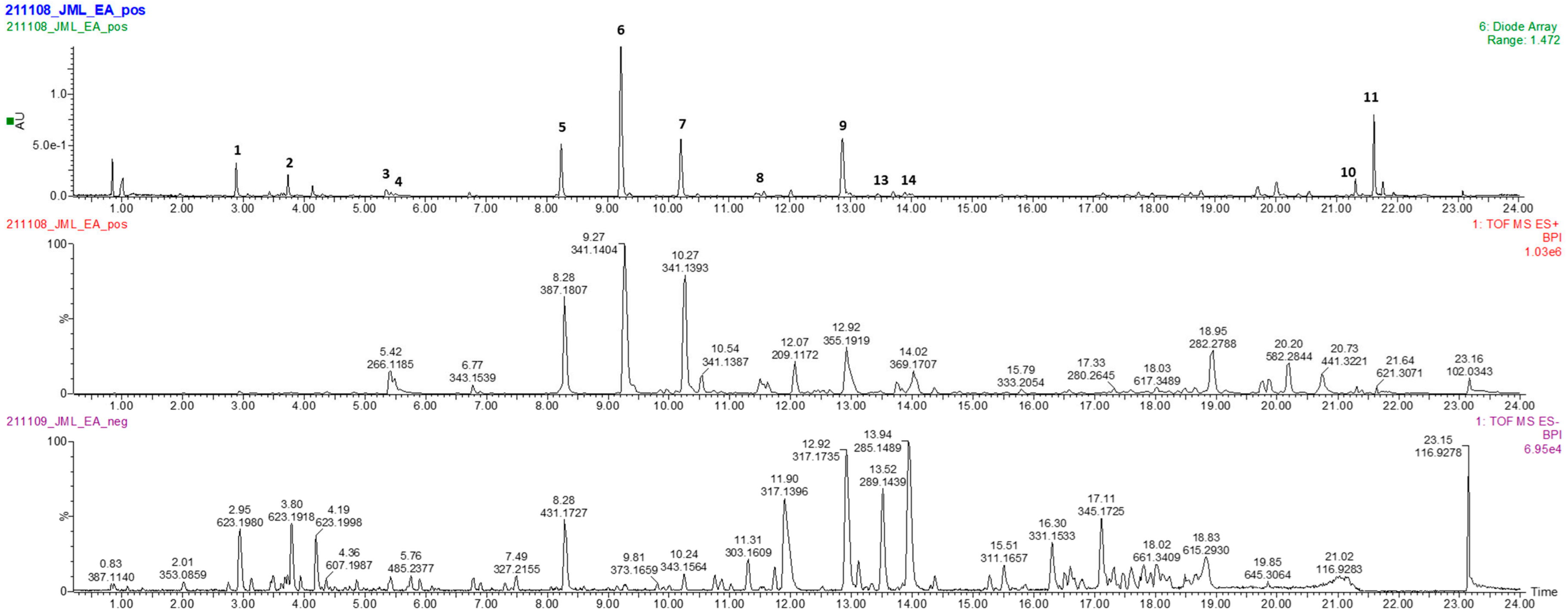

Up: DAD total wavelength chromatogram. Middle: LC-MS base peak ion chromatogram, positive mode, Down: LC-MS base peak ion chromatogram, negative mode.

1: Acteoside, **2**: Acteoside isomer, **3**: Norstephanine, **4**:Anonaine, **5**: Denudatone (**isolated**), **6**: Futoenone (**isolated**), **7**: *cis*-Burchelin (**isolated**), **8**: Nectandrin A, **9**: 14-Deoxy-11,12-didehydroandrographolide, **10**: Pheophytin A, **11**: Pheophorbide A

Figure S30. Molecular network cluster containing lignans

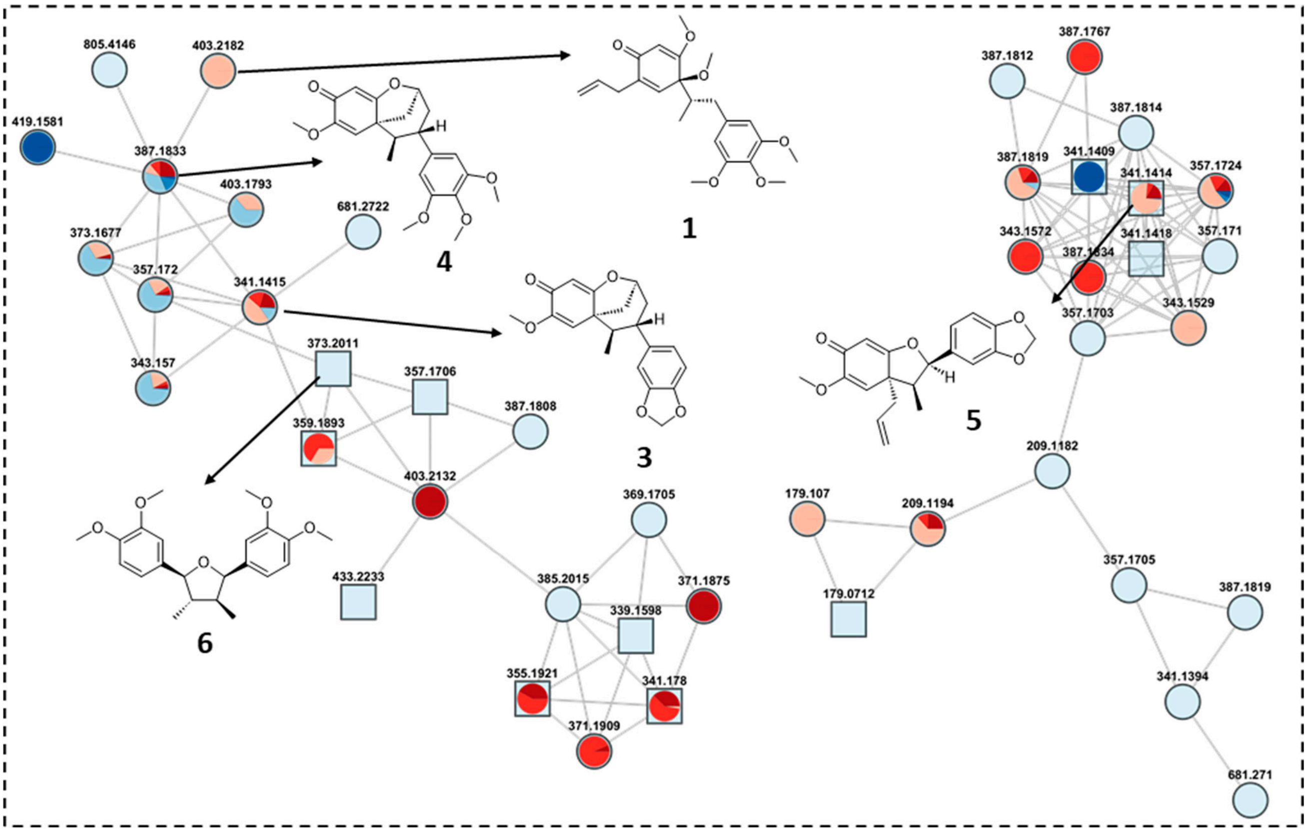

Figure S31. Molecular network cluster containing anonaine, biomarker candidate

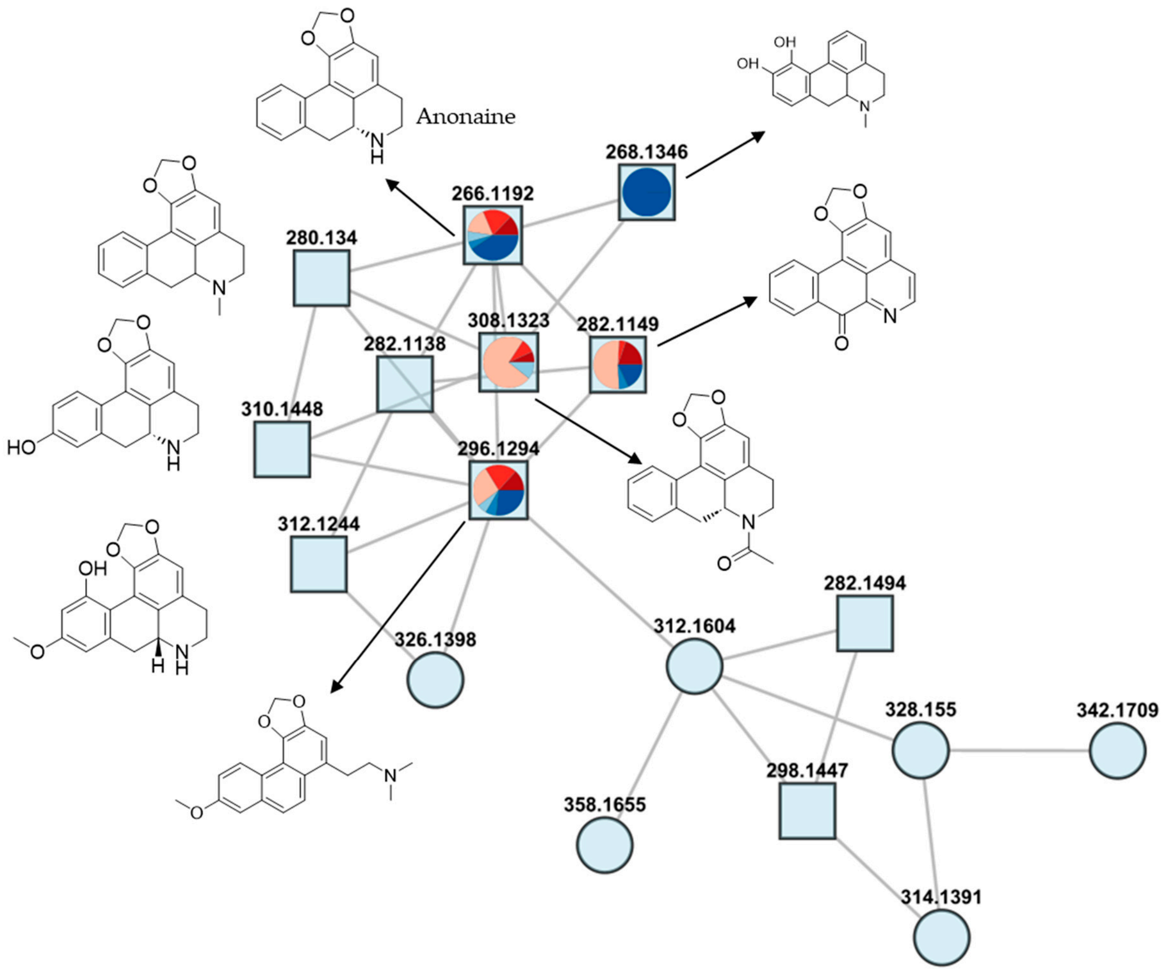

Figure S32. Gene ontology enrichment of bioactive compounds predicted targets (from STRING database).

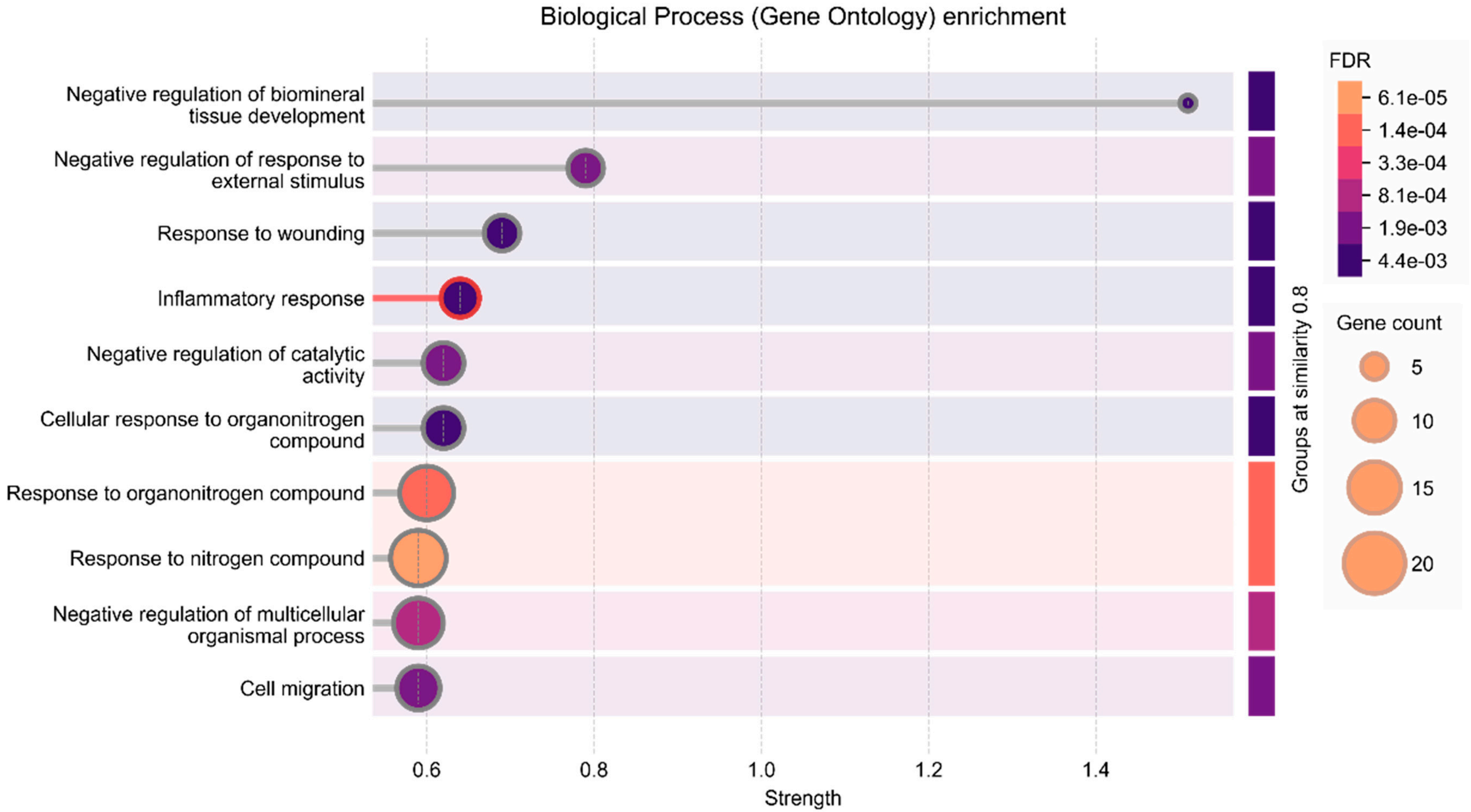

Figure S33. Predicted target interaction network of the isolated active compounds.

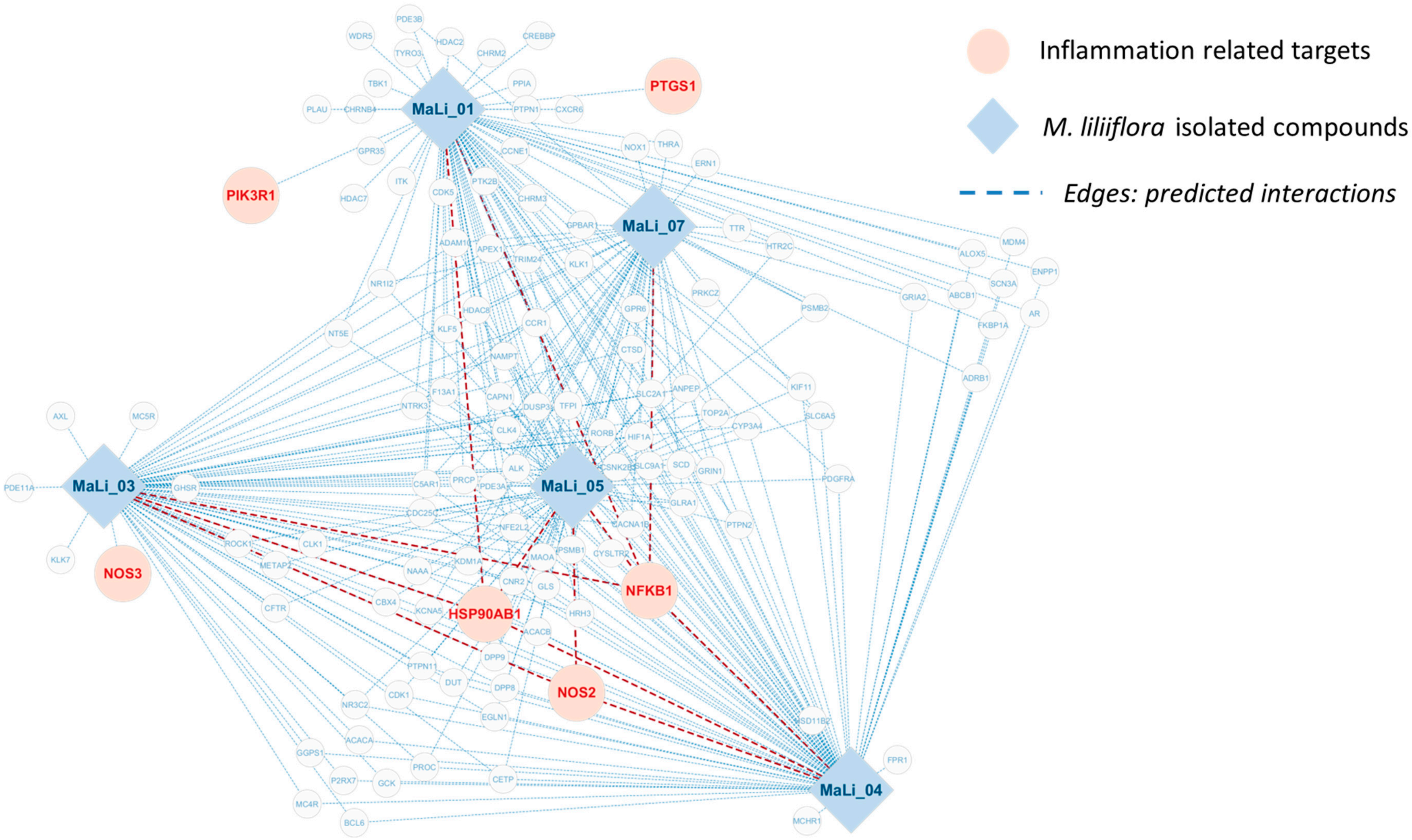

Figure S34. Molecular docking results of compound 1 in predicted targets IKKβ and TAB1-TAK1/2.

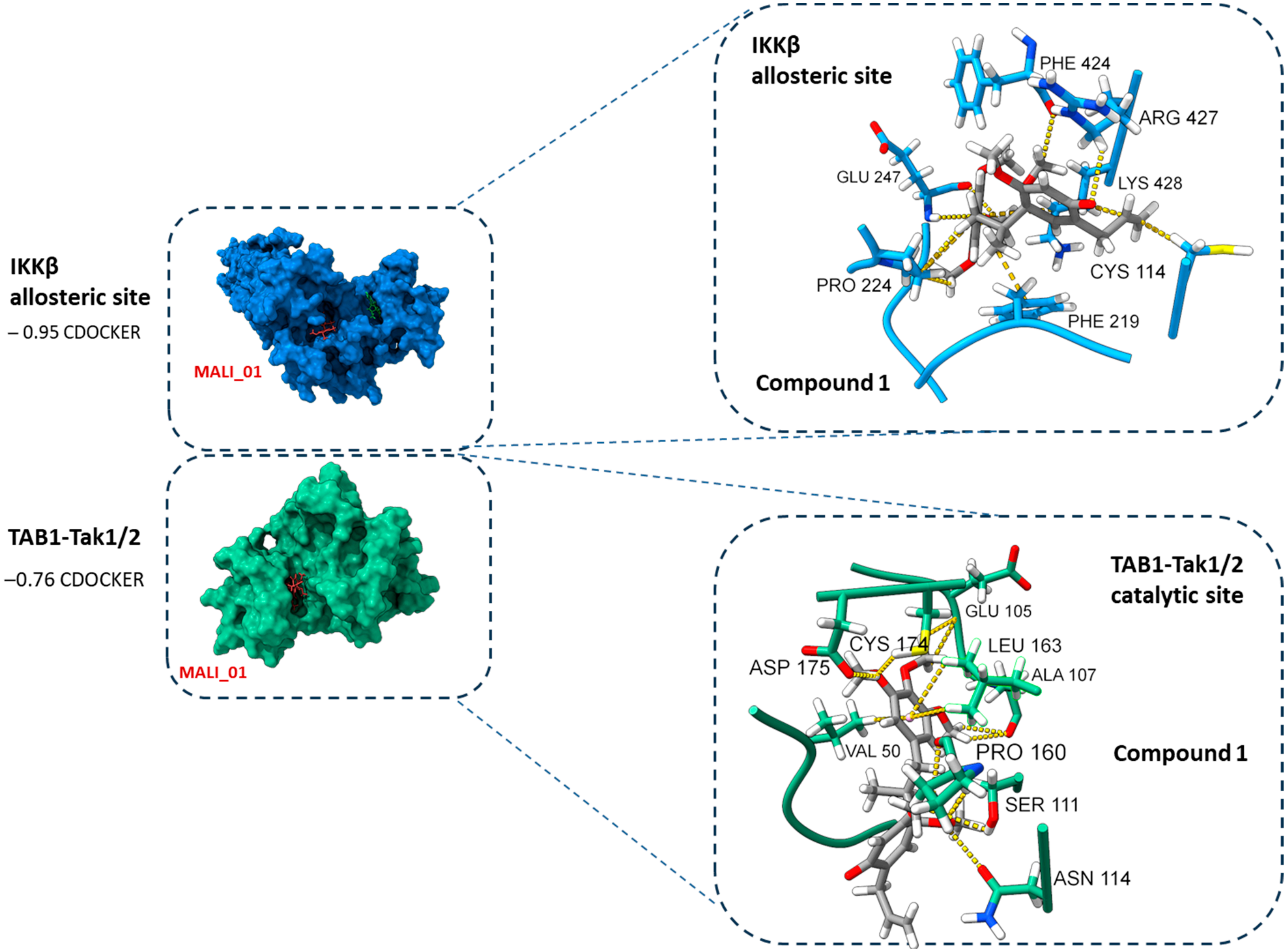

Figure S35. Boiled-Egg diagram representing the predicted permeability of isolated compounds.

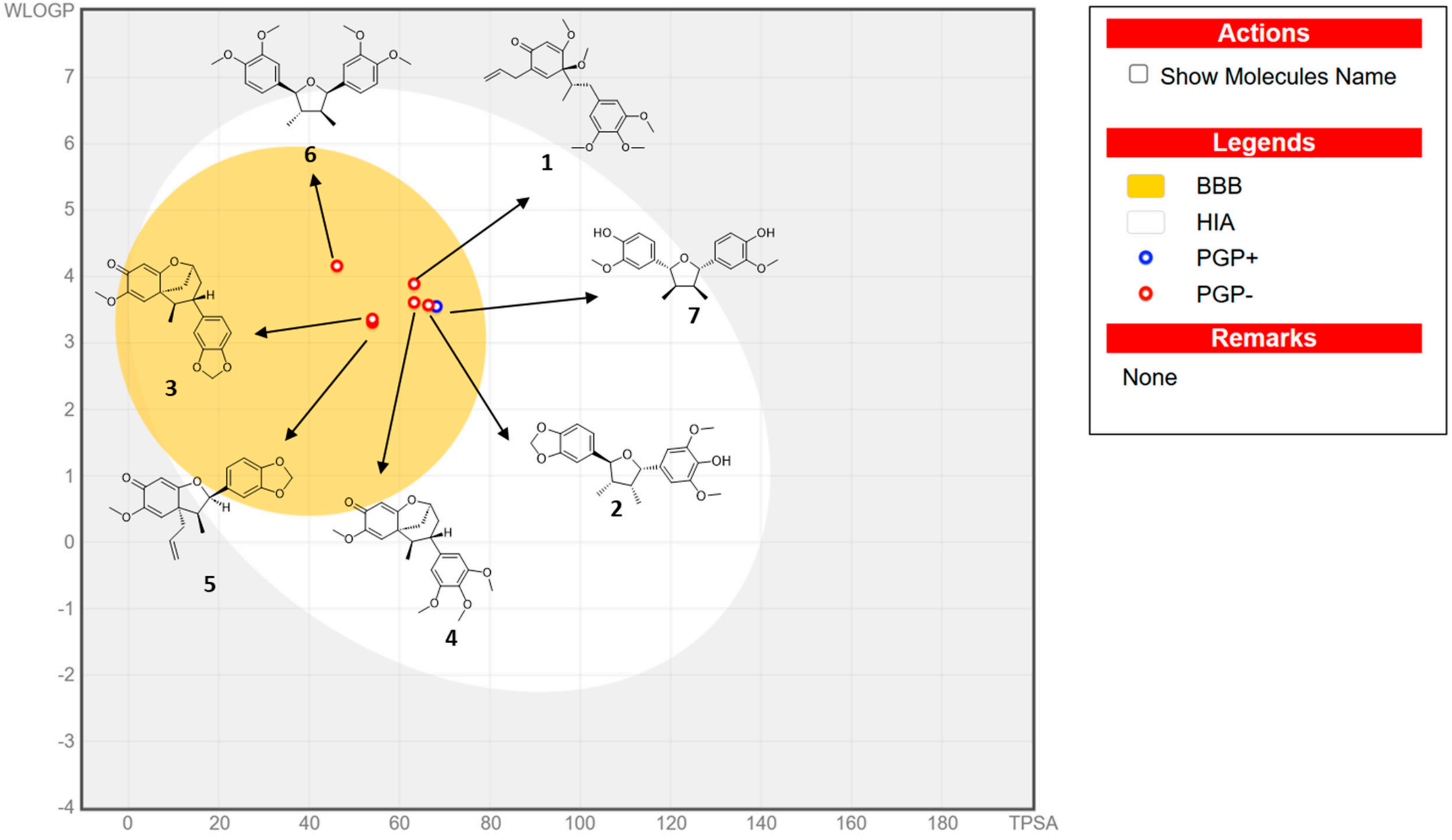

BBB: Blood-brain barrier. HIA: human gastrointestinal absorption. P-gp: premeability glycoprotein

Table S1. Effects of *Magnolia liliiflora* leaf fractions on nitric oxide production in LPS-stimulated BV2 microglial cells

Sample	NO production (μM)
Negative control	2.6 ± 0.6
LPS	87.3 ± 8.2
Curcumin	7.0 ± 0.4
Hexane	59.7 ± 4.0
EtOAc	43.9 ± 2.0
BuOH	34.3 ± 2.7

Nitric oxide (NO) production levels in BV2 microglial cells treated with different solvent fractions of *Magnolia liliiflora* leaves. Cells were pretreated with each fraction (Hexane, EtOAc, and BuOH) at a concentration of 10 μg/mL, followed by stimulation with lipopolysaccharide (LPS, 1 μg/mL) for NO induction. Curcumin (10 μM) was used as a positive control. NO levels were quantified using the Griess reaction. The negative control was not treated with LPS. Data are presented as the mean ± standard deviation (SD).

Table S2. Inhibitory effects of EtOAc subfractions of *Magnolia liliiflora* leaves on nitric oxide production in LPS-stimulated BV2 microglial cells

Sample	NO production (μM)
LPS	26.6 ± 0.8
Negative control (without LPS)	2.7 ± 0.2
Curcumin	2.5 ± 0.1
MaLi. EA.1	4.7 ± 1
MaLi. EA.2	2.6 ± 1.2
MaLi. EA.3	15.1 ± 0.8
MaLi. EA.4	3.3 ± 1.2
MaLi. EA.5	8.2 ± 1.8
MaLi. EA.6	13.3 ± 1.3

Nitric oxide (NO) production levels in BV2 microglial cells treated with EtOAc subfractions (MaLi.EA.1–MaLi.EA.6) of *Magnolia liliiflora* leaves. Cells were pretreated with each subfraction at a concentration of 10 μg/mL, followed by stimulation with lipopolysaccharide (LPS, 1 μg/mL) to induce NO production. Curcumin (10 μM) was used as a positive control, and the negative control was not treated with LPS. NO levels were quantified using the Griess reaction**.** Data are expressed as the mean ± standard deviation (SD).

Table S3. Comparison of nitric oxide inhibitory effects of EtOAc fractions from *Magnolia* species in LPS-stimulated BV2 microglial cells

Sample	NO production (μM)
LPS	80.3 ± 7.5
Negative control (without LPS)	2.7 ± 0.5
Curcumin	8.7 ± 1.1
M. liliiflora	45.4 ± 3.0
M. denudata	58.2 ± 2.7
M. cylindrica	60.1 ± 2.0
M. sprengeri	60.0 ± 0.4
M. acuminata var. Yellow sea	47.6 ± 2.7
M. sargentiniana	63.1 ± 1.7
M. zenii	57.8 ± 1.8

Table S4 Summary of NO inhibition activity of Magnolia species and *Magnolia liliiflora* fractions

**Extract / fraction**	**% inhibition**
Curcumin (positive control)	92 ± 2
M. liliiflora - Hexane	32 ± 3
M. liliiflora - Ethyl Acetate	51 ± 4
M. liliiflora - Buthanol	63 ± 0.3
M. liliiflora - EA.1	92 ± 3
M. liliiflora - EA.2	100 ± 0.3
M. liliiflora - EA.3	48 ± 3
M. liliiflora - EA.4	97 ± 0.5
M. liliiflora - EA.5	77± 4
M. liliiflora - EA.6	55 ± 2
M. denudata - EA	45 ± 5
M. cylindrica - EA	25 ± 5
M. sprengeri - EA	26 ± 5
M. acuminata var. Yellow sea - EA	42 ± 5
M. sargentiniana - EA	22 ± 4
M.zenii - EA	26 ± 6

Table S5. Annotated features of *M. liliiflora* using GNPS library

	$[M+H]^+$ m/z	RT	GNPS annotation	mass difference	Npclassifier superclass	Npclassifier class	Npclassifier pathway
**1**	459.0976	2.8	Epigallocatechin gallate	0.0056	Flavonoids	Flavan-3-ols	Shikimates and Phenylpropanoids
**2**	625.2122	3.0	Acteoside	0.0028	Phenylpropanoids (C6-C3)	Phenylethanoids	Shikimates and Phenylpropanoids
**3**	471.1520	3.1	[2,6-dihydroxy-5-[3,4,5-trihydroxy-6-(hydroxymethyl)oxan-2-yl]oxycyclohex-3-en-1-yl] (E)-3-(3,4-dihydroxyphenyl)prop-2-enoate	0.0030	Phenylpropanoids (C6-C3)	Cinnamic acids and derivatives	Shikimates and Phenylpropanoids
**4**	314.1753	3.2	Armepavine	0.0003	Tyrosine alkaloids	Tetrahydroisoquinoline alkaloids	Alkaloids
**5**	282.1149	3.3	Anolobine	0.0029	Tyrosine alkaloids	Isoquinoline alkaloids	Alkaloids
**6**	193.0866	3.4	4-methoxy-6-prop-2-enyl-1,3-benzodioxole	0.0006	Phenylpropanoids (C6-C3)	Cinnamic acids and derivatives	Shikimates and Phenylpropanoids
**7**	611.1619	3.4	(3,4-dihydroxyphenyl)-5,7-dihydroxy-3-[(2S,3R,4S,5S,6R)-3,4,5-trihydroxy-6-[[(2R,3R,4R,5R,6S)-3,4,5-trihydroxy-6-methyloxan-2-yl]oxymethyl]oxan-2-yl]oxychromen-4-one	0.0020	Flavonoids	Flavonols	Shikimates and Phenylpropanoids
**8**	286.1455	3.6	Coclaurine	0.0015	Tyrosine alkaloids	Tetrahydroisoquinoline	Alkaloids

						alkaloids	
**9**	465.1034	3.7	2-(3,4-dihydroxyphenyl)-5,7-dihydroxy-3-[(2S,3R,4S,5S,6R)-3,4,5-trihydroxy-6-(hydroxymethyl)oxan-2-yl]oxychromen-4-one	0.0014	Flavonoids	Flavonols	Shikimates and Phenylpropanoids
**10**	193.0502	3.8	6-Methoxy-7-hydroxycoumarin	0.0002	Coumarins	Simple coumarins	Shikimates and Phenylpropanoids
**11**	312.1244	3.8	Calycinine	0.0014	Tyrosine alkaloids	Isoquinoline alkaloids	Alkaloids
**12**	471.1516	3.9	[2,6-dihydroxy-5-[3,4,5-trihydroxy-6-(hydroxymethyl)oxan-2-yl]oxycyclohex-3-en-1-yl] (E)-3-(3,4-dihydroxyphenyl)prop-2-enoate	0.0026	Phenylpropanoids (C6-C3)	Cinnamic acids and derivatives	Shikimates and Phenylpropanoids
**13**	325.0939	3.9	Spectral Match to Lactulose from NIST14	0.0171	Saccharides	Disaccharides	Carbohydrates
**14**	268.1346	3.9	Massbank: Apomorphine	0.0006	Tyrosine alkaloids	Isoquinoline alkaloids	Alkaloids
**15**	595.1688	4.0	Massbank:PR301910 Kaempferol-7-O-neohesperidoside	0.0028	Flavonoids	Flavonols	Shikimates and Phenylpropanoids
**16**	625.2121	4.0	[(2R,3R,4S,5R,6R)-6-[2-(3,4-dihydroxyphenyl)ethoxy]-3,5-dihydroxy-4-[(2R,3R,4R,5R,6S)-3,4,5-trihydroxy-6-methyloxan-2-yl]oxyoxan-2-yl]methyl (E)-3-(3,4-dihydroxyphenyl)prop-2-enoate	0.0001	Phenylpropanoids (C6-C3)	Phenylethanoids	Shikimates and Phenylpropanoids

**17**	314.1755	4.1	N,O-dimethylcoclaurine	0.0005	Tyrosine alkaloids	Tetrahydroisoquinoline alkaloids	Alkaloids
**18**	282.1138	4.1	Anolobine	0.0018	Tyrosine alkaloids	Isoquinoline alkaloids	Alkaloids
**19**	449.1084	4.2	(2R,3R)-2-(3,4-dihydroxyphenyl)-3,5,7-trihydroxy-6-[(3R,4R,5S,6R)-3,4,5-trihydroxy-6-(hydroxymethyl)oxan-2-yl]-2,3-dihydrochromen-4-one	0.0014	Flavonoids	Dihydroflavonols	Shikimates and Phenylpropanoids
**20**	197.1177	4.5	Benzofuranone, 5,6,7,7a-tetrahydro-6-hydroxy-4,4,7a-trimethyl-, (6S,7aR)-	0.0007	Apocarotenoids	Apocarotenoids (β-)	Terpenoids
**21**	328.1911	5.0	O-methylarmepavine	0.0001	Tyrosine alkaloids	Tetrahydroisoquinoline alkaloids	Alkaloids
**22**	344.1861	5.0	Anomurine	0.0001	Tyrosine alkaloids	Isoquinoline alkaloids	Alkaloids
**23**	298.1447	5.2	3-hydroxynornuciferine	0.0007	Tyrosine alkaloids	Isoquinoline alkaloids	Alkaloids
**24**	721.3249	5.3	isolariciresinol	0.0029	Lignans	Arylnaphthalene and aryltetralin lignans	Shikimates and Phenylpropanoids
**25**	266.1192	5.4	Anonaine	0.0022	Tyrosine alkaloids	Isoquinoline alkaloids	Alkaloids
**26**	282.1494	5.4	N-nornuciferine	0.0006	Tyrosine alkaloids	Isoquinoline alkaloids	Alkaloids
**27**	280.1340	5.5	roemerine	0.0040	Tyrosine alkaloids	Isoquinoline alkaloids	Alkaloids
**28**	296.1294	5.5	Norstephanine	0.0014	Tyrosine alkaloids	Isoquinoline alkaloids	Alkaloids
**29**	310.1448	5.6	Stephanine	0.0008	Tyrosine alkaloids	Isoquinoline alkaloids	Alkaloids
**30**	303.0510	6.0	Massbank:PR302812 Quercetin	0.0010	Flavonoids	Flavonols	Shikimates and Phenylpropanoids

**31**	358.2014	6.1	N-methylanomurine	0.0006	Tyrosine alkaloids	Isoquinoline alkaloids	Alkaloids
**32**	419.1696	6.2	4-[(3R,3aR,6S,6aR)-6-(4-hydroxy-3,5-dimethoxyphenyl)-1,3,3a,4,6,6a-hexahydrofuro[3,4-c]furan-3-yl]-2,6-dimethoxyphenol	0.0004	Lignans	Furofuranoid lignans	Shikimates and Phenylpropanoids
**33**	837.3323	6.2	4-[(3R,3aR,6S,6aR)-6-(4-hydroxy-3,5-dimethoxyphenyl)-1,3,3a,4,6,6a-hexahydrofuro[3,4-c]furan-3-yl]-2,6-dimethoxyphenol	0.0033	Lignans	Furofuranoid lignans	Shikimates and Phenylpropanoids
**34**	287.0565	7.2	Kaempferol|3,5,7-trihydroxy-2-(4-hydroxyphenyl)chromen-4-one	0.0015	Flavonoids	Flavonols	Shikimates and Phenylpropanoids
**35**	317.0671	8.7	Rhamnetin	0.0011	Flavonoids	Flavonols	Shikimates and Phenylpropanoids
**36**	369.1701	9.4	4-[(1R,5R)-6-(3,4-dimethoxyphenyl)-3,7-dioxabicyclo[3.3.0]oct-2-yl]-1,2-dimeth oxybenzene	0.0001	Lignans	Furofuranoid lignans	Shikimates and Phenylpropanoids
**37**	231.1385	9.5	6-Hydroxy-5a-methyl-3,9-bis(methylene)decahydronaphtho[1,2-b]furan-2(3H)-one	0.0015	Sesquiterpenoids	Germacrane sesquiterpenoids	Terpenoids
**38**	249.1489	9.5	reynosin	0.0001	Sesquiterpenoids	Germacrane sesquiterpenoids	Terpenoids
**39**	233.1541	9.6	(+)-Costunolide	0.0001	Sesquiterpenoids	Germacrane sesquiterpenoids	Terpenoids
**40**	203.1802	9.7	b-Caryophyllene oxide	0.0002	Sesquiterpenoids	Caryophyllane sesquiterpenoids	Terpenoids

**41**	417.1898	9.7	Magnolin	0.0002	Lignans	Furofuranoid lignans	Shikimates and Phenylpropanoids
**42**	205.1958	9.9	1,7-Dimethyl-7-(4-methyl-3-penten-1-yl)bicyclo[2.2.1]heptan-2-ol	0.0008	Sesquiterpenoids	Campherenane sesquiterpenoids	Terpenoids
**43**	447.2018	10.0	epiyangambin	0.0008	Lignans	Furanoid lignans	Shikimates and Phenylpropanoids
**44**	893.3941	10.0	3,6-bis(3,4,5-trimethoxyphenyl)-1,3,3a,4,6,6a-hexahydrofuro[3,4-c]furan	0.0026	Lignans	Furofuranoid lignans	Shikimates and Phenylpropanoids
**45**	341.1414	10.2	(2R,3S,3aS)-2-(1,3-benzodioxol-5-yl)-5-methoxy-3-methyl-3a-prop-2-enyl-2,3-dihydro-1-benzofuran-6-one	0.0024	Lignans	Neolignans	Shikimates and Phenylpropanoids
**46**	308.1323	10.4	N-acetylanonaine	0.0043	Tyrosine alkaloids	Isoquinoline alkaloids	Alkaloids
**47**	401.1607	10.8	aschantin	0.0017	Lignans	Furofuranoid lignans	Shikimates and Phenylpropanoids
**48**	341.1409	10.8	(2R,3S,3aS)-2-(1,3-benzodioxol-5-yl)-5-methoxy-3-methyl-3a-prop-2-enyl-2,3-dihydro-1-benzofuran-6-one	0.0019	Lignans	Neolignans	Shikimates and Phenylpropanoids
**49**	341.1780	11.4	4-[5-(3,4-Dimethoxyphenyl)-3,4-dimethyltetrahydro-2-furanyl]-2-methoxyphenol	0.0040	Lignans	Furanoid lignans	Shikimates and Phenylpropanoids
**50**	359.1893	11.4	4-[5-(3,4-Dimethoxyphenyl)-3,4-dimethyltetrahydro-2-furanyl]-2-	0.0043	Lignans	Furanoid lignans	Shikimates and Phenylpropanoids

			methoxyphenol				
**51**	179.0712	11.5	Coniferyl aldehyde	0.0012	Phenylpropanoids (C6-C3)	Cinnamic acids and derivatives	Shikimates and Phenylpropanoids
**52**	341.1418	11.6	(2R,3S,3aS)-2-(1,3-benzodioxol-5-yl)-5-methoxy-3-methyl-3a-prop-2-enyl-2,3-dihydro-1-benzofuran-6-one	0.0028	Lignans	Neolignans	Shikimates and Phenylpropanoids
**54**	373.2094	12.5	Galgravin	0.0134	Lignans	Furanoid lignans	Shikimates and Phenylpropanoids
**55**	355.1921	12.9	14-Deoxy-11,12-didehydroandrographolide	0.0121	Diterpenoids	Labdane diterpenoids	Terpenoids
**56**	373.2011	12.9	Veraguensin	0.0011	Lignans	Furanoid lignans	Shikimates and Phenylpropanoids
**57**	433.2233	13.0	3,4-dimethyl-2,5-bis(3,4,5-trimethoxyphenyl)oxolane	0.0013	Lignans	Furanoid lignans	Shikimates and Phenylpropanoids
**58**	163.0761	13.5	3,4-Methylenedioxyamphetamine	0.0011	Alkaloids	Alkaloids	Alkaloids
**59**	357.1706	14.0	4-[1-(1,3-benzodioxol-5-yl)propan-2-yl]-4,5-dimethoxy-2-prop-2-enylcyclohexa-2,5-dien-1-one	0.0006	Lignans	Arylnaphthalene and aryltetralin lignans	Shikimates and Phenylpropanoids
**60**	339.1598	14.1	4-[1-(1,3-benzodioxol-5-yl)propan-2-yl]-4,5-dimethoxy-2-prop-2-enylcyclohexa-2,5-dien-1-one	0.0018	Lignans	Neolignans	Shikimates and Phenylpropanoids
**61**	137.1334	15.5	Citronellal	0.0011	Monoterpenoids	Acyclic monoterpenoids	Terpenoids
**62**	205.1960	15.7	a-Bisabolol	0.0000	Sesquiterpenoids	Bisabolane sesquiterpenoids	Terpenoids

**63**	353.2699	16.7	Monolinolenin (9c,12c,15c)	0.0001	Glycerolipids	Monoacvylglycerol	Fatty acids
**64**	279.2328	18.0	Linolenic acid	0.0008	Fatty Acids and Conjugates	Unsaturated fatty acids	Fatty acids
**65**	511.5199	18.5	amylamine-C11:0	0.0001	Fatty amides	N-acyl amines	Fatty acids
**66**	609.2719	18.7	Hydroxypheophorbide a	0.0001	Tryptophan alkaloids		Alkaloids
**67**	282.2799	19.0	9-Octadecenamide	0.0009	Fatty amides	Primary amides	Fatty acids
**68**	593.2761	19.8	Phaeophorbide a	0.0021	Tryptophan alkaloids	Carboline alkaloids	Alkaloids
**69**	535.2701	20.5	Pyropheophorbide a	0.0001	Tryptophan alkaloids	Carboline alkaloids	Alkaloids
**70**	871.5855	21.4	Pheophytin a	0.0145	Meroterpenoids	Prenyl quinone meroterpenoids	Terpenoids
**71**	696.5533	21.4	GalCer(d18:2/16:1)	0.0118	Sphingolipids	Neutral glycosphingolipids	Fatty acids

Table S6. Biomarkers candidate annotation

m/z	RT (min)	Correlation (Spearman)	*p*-value	FDR	Annotation	Annotation method
387.1833	8.27	0.87	0.00	0.01	Futoenone	Isolation
343.1570	6.85	0.79	0.00	0.02	*Lignan	Molecular network
341.1415	9.20	0.79	0.00	0.02	Denudatone	Isolation
266.1192	5.41	0.71	0.01	0.03	Anonaine	GNPS MS/MS match
233.0825	9.05	0.70	0.01	0.04	Non annotated	-
278.0836	4.08	0.69	0.01	0.04	Non annotated	-

Table S7. Molecular docking results on Inflammatory related targets (-CDOCKER values)

Protein	Binding site	PDB	1	3	4	5	7
NF-kB (p50)	DNA binding site	1SVC	-	-	-	-	-
NF-kB (p65-p50)	DNA binding site	1NFI	-10.57	-31.63	-17.32	-29.73	17.15
NF-kB (p65-p50-IkB)	p65-NFkB interaction	1IKN	-12.65	-31.01	-20.64	-28.3	11.31
IKKb	catalytic site	4KIK protomer A	-4.94	-25.95	-13.39	-23.56	28.12
IKKb	allosteric site	4KIK protomer A	-3.37	-22.04	-14.92	-22.27	24.09
IKKb	allosteric site	4KIK protomer B	-0.95	-23.61	-12.5	-24.29	19.84
TAB1 - TAK1/2	active site	9FPD	-0.76	-25.77	-15.47	-22.78	27.15
HSP90	ATP active site	5UC4	-3.36	-24.16	-10.18	-20.66	21.14
iNOS	active site	3E7G	-8.34	-24.76	-18.74	-24.95	25.47
COX	active site	5F1A	-	-	-	-	18.54
